# Sociodemographic, behavioral, and medical risk factors associated with visual impairment among older adults: a community-based pilot survey in Southern District of Hong Kong

**DOI:** 10.1186/s12886-020-01644-1

**Published:** 2020-09-18

**Authors:** Perseus Wing-Fu WONG, Jordy Kin-Pong LAU, Bonnie Nga-Kwan CHOY, Kendrick Co SHIH, Alex Lap-Ki NG, Ian Yat-Hin WONG, Jonathan Cheuk-Hung CHAN

**Affiliations:** Department of Ophthalmology, LKS Faculty of Medicine, University of Hong Kong, Room 301, Block B, Cyberport 4, Pok Fu Lam, Hong Kong

**Keywords:** Visual impairment, Prevalence, Risk factors, Survey, Hong Kong, Disparities, Stratified analysis, Eye-level analysis, Interaction effect

## Abstract

**Background:**

The last visual survey of older adults in Hong Kong was a district-level study in 2002, with no assessment of behavioral and medical risk factors for visual impairment (VI). Our objectives were to determine the latest VI prevalence among older adults, significance of any spatial and temporal differences on the prevalence, and any associations of sociodemographic, behavioral and medical risk factors with VI from a multi-perspective analysis.

**Methods:**

Community-based pilot survey of residents from a suburb of Hong Kong, aged ≥50, using a standardized questionnaire, was conducted in 2016.

**Results:**

Of the 222 subjects, crude rates of bilateral and unilateral VI were 9.46 and 32.88%, respectively, or corresponding age-and-gender-adjusted rates of 6.89 and 30.5%. Older age and lower educational were associated with higher risk for unilateral VI, while older age, temporary housing, obesity and hyperlipidemia were associated with higher risk for bilateral VI. Smoking and alcohol-drinking status were not associated with unilateral or bilateral VI. Relative changes in ORs of hypertension or educational level on unilateral or bilateral VI were >  10% after adjusting for age. Interaction term between hyperlipidemia and gender or obesity was significant for unilateral VI. Gender, hypertension and cataract were not associated with unilateral or bilateral VI in general population of pooled analysis but were identified as risk factors in specific subgroups of stratified analysis. Refractive error (myopia or hyperopia) was significantly associated with VI in the eye-level analysis after adjusting the inter-eye correlation.

**Conclusions:**

Sociodemographic and medical risk factors contributed to VI, but behavioral risk factors did not. Sociodemographic disparities of visual health existed. Age was the confounders of the VI-hypertension or VI-educational level relationships. Gender and obesity were more likely to have multiplicative effect on unilateral VI when combined with hyperlipidemia. Stratified analysis should be conducted to provide further insight into the risk factors for VI in specific populations. Uncorrected refractive error remains a significant cause of impaired vision. The spatial and temporal differences in bilateral VI prevalence from the previous local study indicates a territory-wide survey is needed to assess regional differences and overall prevalence of VI in Hong Kong.

## Introduction

### Background

Visual impairment (VI) is significantly associated with poor physical health, limitation, and disability [1, 2]. Previous studies have shown a correlation between the severity of VI and the incidence of falls, fractures, chronic diseases and mortality in the elderly [3–8]. Visually impaired people are more likely to have poorer social network, lower employment status, and mental health problems [9–11]. Using different scoring scales to measure and evaluate quality of life (QoL) have suggested that VI severity is negatively correlated with QoL scores [12–17]. Increased VI is also correlated with substantial health burden [18]. Elderly people with VI are more likely to require social support services and nursing home placement, [19, 20] and their community would also need to bear the associated medical cost and productivity loss [21, 22].

Socioeconomic factors can explain 69.4% of the global variations in the prevalence of moderate to severe VI and 76.3% of the global variations in prevalence of blindness [23]. Unfavorable socioeconomic status is associated with a higher prevalence of low vision and blindness in many countries [24–28]. Behavioral factors like smoking status and alcohol-drinking pattern were associated with VI among older adults, [29, 30] while biomarkers such as increased serum low-density lipoprotein (LDL), homocysteine, and being underweight were shown to be associated with blindness [31]. Thus, individual health is mutually determined by the biological, behavioral, cultural, social and environmental factors. A broader framework was required for analyzing visual health problems from biosocial and socio-behavioral perspectives [32].

### Rationales and objectives

To our knowledge, there was no territory-wide visual health survey of older adults in Hong Kong but had been a district-level survey in 2002, in the Shatin District (a northern region of Hong Kong which is less densely populated than the main urban areas of Hong Kong Island and Kowloon) [33]. We hypothesized that spatial (Shatin and Southern Districts) and temporal (2002 and 2016) differences on the prevalence of VI among older adults would be statistically significant at district-level of Hong Kong when compared our results with the previous survey in 2002.

In addition, the survey in 2002 only covered the sociodemographic characteristics of the subjects in statistical analysis, but did not measure the associations of behavioral and medical risk factors with VI. The Singapore Malays Eye Study was an example of examining the associations of sociodemographic, behavioral and medical risk factors with VI, which shown that older age, lower education, unemployment, being widowed, diabetes and hypertension were independently associated with bilateral VI [34]. We hypothesized that behavioral and medical risk factors would be associated with VI among older adults, other than sociodemographic characteristics.

Our primary objective was to estimate the prevalence of VI among older adults within Southern District (located on the southern part of Hong Kong Island), in order to examine the significance of spatial and temporal differences on the prevalence of VI at district-level of Hong Kong when compared our results with the survey in 2002 and then show the rationale of conducting a territory-wide survey throughout Hong Kong.

The secondary objective was to measure the associations of sociodemographic, behavioral and medical risk factors with VI among older adults, in order to review the visual health problems and identify the vulnerable groups for VI from biosocial and socio-behavioral perspectives. The findings of this pilot survey will be useful in evaluating the feasibility of a forthcoming 5-year eye screening programme, and designing future large-scale eye research surveys.

## Methods

### Ethics approval and informed consent

Ethics approval of this study was granted by the Institutional Review Board (IRB) and Ethics Committees (EC) of the Hong Kong University / Hospital Authority Hong Kong West Cluster (reference number: UW 15–160) on 16th March 2015. All subjects agreeing to participate in the study gave their written, informed consent prior to the administration of any eye assessment or questionnaire.

### Study area and sampling

This community-based, cross-sectional, pilot survey was conducted at Chi Fu, which is a suburb randomly selected from the 17 possible District Council Constituency Areas (DCCAs) of the Southern District of Hong Kong. According to the 2016 Population By-census, Chi Fu had an estimated population of 15,784 and domestic households of 5437. The residents aged 50 and above for this area was 6427 (46.52% males, 53.48% females) [35]. On average, its residents had a higher educational level, household income, and proportion living in private housing (rather than public housing) than the mean for the entire Southern District, Shatin District and Hong Kong. (Supplementary file 1, Table 6).

Invitation letters (in Chinese) for a free eye assessment and visual health survey, to be conducted at the local community hall, were sent to all registered households within the study area (i.e. Chi Fu DCCA) at the same time to ensure that all residents would have the same opportunity to get the information and participate in our survey. On a first-come-first-served basis (i.e. convenience sampling which is common in pilot study), residents who were interested to participate responded by calling back to make an appointment at a designated time for a free eye consultation with an ophthalmologist, during which, trained research assistants explained the details of the study and distributed the questionnaire (Supplementary files 10, 11) for their own completion. The targeted study population were local Chinese residents aged 50 and above, with no extra inclusion and exclusion criteria. The survey fieldwork was carried out from the 11th to the 28th, January 2016.

### Measurements

At the study site, optometrists measured the presenting visual acuity (VA) for each eye of the subject, using a Logarithm of the Minimum Angle of Resolution (logMAR) visual acuity chart at a distance of 3 m. If none of the large optotype on the highest line could be identified, VA was then assessed for counting fingers, hand motion, and presence or absence of light perception, in that order. Refractive error was measured by subjective refraction. Experienced ophthalmic assistants measured intraocular pressure and performed autorefraction using NIDEK Tonoref II. Slip-lamp examination of eyelids, ocular surface and anterior segment using Topcon SL 701 with DC4, as well as optical coherence tomography (OCT) scan using the Cirrus-5000 SD-OCT were performed by ophthalmologists, to examine the fundus without dilating the pupil and measure the macula and peripapillary retinal nerve fiber layer (RNFL) thickness and volume. We did not test the visual field defects, but measured the blood pressure, ankle-brachial index (ABI), body weight, and height of the subjects.

A structured and self-administrated questionnaire in Chinese was used to collect data on sociodemographic, behavioral, and medical characteristics. Sociodemographic and behavioral information covered age, gender, type of housing, educational level, marital status, employment status, monthly household income, smoking and alcohol-drinking habits. Subjects were inquired for any history of chronic medical conditions like diabetes mellitus (DM), hypertension, and hyperlipidemia, as well as three common eye diseases including age-related macular degeneration (AMD), cataract, and glaucoma.

### Operational definitions

We used the World Health Organization (WHO) criteria to define blindness as presenting VA of < 3/60 (logMAR > 1.3) and low vision as presenting VA of < 6/18 and ≥ 3/60 (logMAR > 0.5 and ≤ 1.3), without considering the visual fields [36]. Bilateral VI included both blindness and low vision in the better-seeing eye. For providing further insight into unilateral VI, we presented the results of blindness and low vision with the same definition in terms of the worse-seeing eye [34, 37]. Myopia and hyperopia were defined as spherical equivalent refractions of <− 0.50 diopter or > + 0.50 diopter, respectively [38]. Body Mass Index (BMI) is equivalent to the weight (in kilograms) over height squared (in centimeters) and was categorized into underweight (< 18.5), normal weight (18.5–22.9), overweight (23–24.9), and obesity (≥25) [39–41]. Ankle-brachial index (ABI) was categorized into blockage (< 1.0), normal (1.0–1.4), and rigid arteries (> 1.4) [42]. Blood pressure was categorized into low (≤90/60), normal (> 90/60 and < 120/80), pre-high (120/80–139/89), and high (≥140/90) [43, 44].

### Data analysis

For descriptive data analysis, median with interquartile range (IQR) was used to summarize continuous variables, whilst frequencies and percentages were used to summarize categorical variables. Crude, age-and-gender-adjusted, age-specific and gender-specific prevalence estimates of low vision, blindness, bilateral and unilateral VI were expressed in percentage with 95% confidence intervals (CIs). Relative changes in odds ratios (ORs) were calculated to measure the magnitude of confounding and examine the confounding effect of age on the associations of VI with obesity, hypertension, hyperlipidemia and educational level. Multiplicative interaction model and stratified analysis were done to examine the effect modification between independent variables on the risk for unilateral and bilateral VI. Univariate and multivariate logistic models with backward stepwise method were conducted to examine the associations between VI and independent variables at subject-level. Generalized linear mixed effects models (GLMM) were used to investigate the associations of VI with eye examination results at eye-level analysis after adjusting the inter-eye correlation. Crude and adjusted odds ratios (CORs and AORs) with 95% CIs were calculated to show the strength of associations. A *p*-value of < 0.05 was considered statistically significant. We included records with incomplete data in analyses and used multiple imputation method to handle missing data by creating 2000 multiple datasets and combining estimates from imputed datasets to obtain the overall estimate, based on the assumption of missing at random [45, 46]. The database was maintained and managed using Microsoft Excel 2016 (Microsoft Corporation, Redmond, WA, USA). All statistical analysis were performed using R package version 3.5.1 (R Development Core Team, 2018) [47].

## Results

### Characteristics of subjects

A total of 222 subjects, median age 67 years (IQR: 61–72), agreed to participate in the study, including 87 males (39.19%) and 135 females (60.81%). The majority was aged between 60 and 69 (49.55%). Most of them had completed secondary education (51.35%) and lived in private housing (94.14%). They were predominantly married (85.14%) and not working (89.64%). Nearly three-fourths (75.22%) had monthly household income below $25,000. The self-reported prevalence of DM, hypertension, hyperlipidemia, AMD, cataract and glaucoma were 12.61, 39.64, 15.32, 4.05, 22.97 and 2.70%, respectively. The proportions of current and past smokers were 1.35 and 3.6%, respectively, whilst those of current and past alcohol-drinkers were 17.12 and 1.8%, respectively. (Supplementary file 2. Table 7).

### Descriptive and univariate logistic analysis

The numbers of right eye and left eye with VI (low vision or blindness) were 46 and 48 respectively whilst the cases of bilateral and unilateral VI were 21 and 73 respectively. The crude rates of bilateral and unilateral VI were 9.46 and 32.88% respectively while the age-and-gender-adjusted rates were 6.89 and 30.5% respectively. Age-specific rates of 59–59, 60–69, 70–79 and ≥ 80 were 17.5, 31.82, 37.74 and 57.89% respectively for unilateral VI, as well as 0, 9.09, 13.21 and 21.05% respectively for bilateral VI. Gender-specific rates of males and females were 34.48 and 31.85% respectively for unilateral VI, as well as 12.64 and 7.41% respectively for bilateral VI. (Supplementary file 3. Table 8).

Univariate logistic regression results showed the significant associations of unilateral or bilateral VI with older age, low educational level and obesity. Household income and having a history of hypertension were significantly associated with unilateral VI, while living in temporary housing and having a history of hyperlipidemia were significantly associated with bilateral VI. (Supplementary file 4. Table 9) We did not find any significant crude association of unilateral or bilateral VI with current or past smoking or alcohol-drinking, but found that current or past smoking was associated with being obese and having a history of hypertension. (Supplementary file 5. Table 10).

### Multivariate logistic analysis

Multivariate logistic regression results with backward stepwise method in Table 1 noted that subjects with older age (AOR 1.04; 95% CI 1.00–1.09) and educational level higher than secondary with either non-degree (AOR 0.28; 95% CI 0.09–0.80) or degree tertiary education (AOR 0.29; 95% CI 0.08–0.89), were significantly associated with unilateral VI. In addition, subjects with older age (AOR 1.08; 95% CI 1.02–1.15), being obese (AOR 4.00; 95% CI 1.49–11.41), having a history of hyperlipidemia (AOR 3.60; 95% CI 1.13–10.97), and living in temporary housing (AOR 8.26; 95% CI 1.81–34.92), were significantly associated with bilateral VI. Older age was likely to be common variable contributing to unilateral or bilateral VI. Our results did not find any significant adjusted association of smoking and alcohol-drinking status with unilateral or bilateral VI.

**Table 1:** Epidemiologic factors associated with visual impairment by the 222 respondents from a southern suburb of Hong Kong in January 2016: Multivariate logistic regression

Variables	Unilateral Visual Impairment		Bilateral Visual Impairment		
	AOR (95% CI)	*p*-value	AOR (95% CI)	*p*-value	
Age (Years)	1.04 (1.00–1.09)	0.031**	1.08 (1.02–1.15)	0.011**	
Educational level:					
Primary level or below (ref)	1.00	–			
Secondary level	0.54 (0.26–1.13)	0.103			
Non-degree level	0.28 (0.09–0.80)	0.022**			
Degree level	0.29 (0.08–0.89)	0.039**			
Housing type:					
Private housing (ref)			1.00	–	
Temporary housing			8.26 (1.81–34.92)	0.004***	
Obesity (BMI ≥ 25)					
No (Ref)	1.00	–	1.00	–	
Yes	1.86 (0.99–3.51)	0.053*	4.00 (1.49–11.41)	0.007	***
Hyperlipidemia					
No (Ref)			1.00	–	
Yes			3.60 (1.13–10.97)	0.025**	
Glaucoma					
No (Ref)	1.00	–			
Yes	4.11 (0.69–32.92)	0.132			

*AOR* adjusted odds ratio, *CI* confidence interval

* *p*-value < 0.1; **p-value < 0.05; *** *p*-value < 0.01

### Confounding, effect modification and stratified analysis

The relative changes in ORs of exposures on unilateral or bilateral VI after adjusting for age were calculated to measure the magnitude of confounding in Table 2. After adjusting for age, for unilateral VI, ORs of having a history of hypertension decreased from 1.97 (1.12–3.51) to 1.52 (0.83–2.79), having non-degree tertiary education increased from 0.2 (0.07–0.56) to 0.26 (0.08–0.72), and having degree tertiary education increased from 0.21 (0.06–0.61) to 0.29 (0.08–0.86), with the effect of age remaining significant. Similarly, for bilateral VI, ORs of having secondary education increased from 0.19 (0.06–0.53) to 0.25 (0.08–0.74). Since the corresponding relative changes in ORs were 22.84, 30, 38 and 31.58%, we noted that age was the confounders of the unilateral VI-hypertension, unilateral VI-educational level and bilateral VI-educational level relationships due to a relative change of > 10% in the ORs of having a history of hypertension or higher educational level on unilateral or bilateral VI after adjusting for age.

**Table 2:** Magnitude of confounding of obesity, hypertension, hyperlipidemia and educational levels on visual impairment after adjusting the age

	Unilateral Visual Impairment		Bilateral Visual Impairment	
	Estimate (95% CI)	p-value	Estimate (95% CI)	*p*-value
A				
Association of age with VI	1.06 (1.02–1.10)	0.001***	1.09 (1.03–1.15)	0.003***
Association of age with obesity	1.03 (0.99–1.06)	0.163	1.03 (0.99–1.06)	0.163
Association of obesity with VI	2.00 (1.09–3.63)	0.024**	3.63 (1.46–9.36)	0.006***
Association of age and obesity with VI				
-Age	1.06 (1.02–1.10)	0.002***	1.09 (1.03–1.16)	0.003***
-Obesity	1.89 (1.02–3.49)	0.041**	3.58 (1.40–9.52)	0.008***
Magnitude of confounding: relative change in OR of obesity on VI after adjusting the age				
- Obesity	5.50%	(< 10%)	1.38%	(< 10%)
B				
Association of age with VI	1.06 (1.02–1.10)	0.001***	1.09 (1.03–1.15)	0.003***
Association of age with history of hypertension	1.10 (1.06–1.14)	< 0.001***	1.10 (1.06–1.14)	< 0.001***
Association of history of hypertension with VI	1.97 (1.12–3.51)	0.019**	2.19 (0.89–5.61)	0.091*
Association of age and history of hypertension with VI				
-Age	1.05 (1.01–1.09)	0.002***	1.08 (1.02–1.15)	0.008***
-History of hypertension	1.52 (0.83–2.79)	0.008**	1.48 (0.56–3.95)	0.426
Magnitude of confounding: relative change in OR of history of hypertension on VI after adjusting the age				
- History of hypertension	22.84%	(> 10%)	32.42%	(> 10%)
C				
Association of age with VI	1.06 (1.02–1.10)	0.001***	1.09 (1.03–1.15)	0.003***
Association of age with history of hyperlipidemia	1.04 (0.99–1.08)	0.121	1.04 (0.99–1.08)	0.121
Association of history of hyperlipidemia with VI	1.53 (0.71–3.22)	0.266	3.22 (1.13–8.51)	0.021**
Association of age and history of hyperlipidemia with VI				
-Age	1.06 (1.02–1.10)	0.002***	1.09 (1.03–1.16)	0.004***
-History of hyperlipidemia	1.37 (0.63–2.93)	0.419	2.96 (1.01–8.06)	0.038**
Magnitude of confounding: relative change in OR of history of hyperlipidemia on VI after adjusting the age				
- History of hyperlipidemia	10.46%	(≈ 10%)	8.07%	(< 10%)
D				
Association of age with VI	1.06 (1.02–1.10)	0.001***	1.09 (1.03–1.15)	0.003***
Association of age with educational level	0.91 (0.88–0.95)	< 0.001***	0.91 (0.88–0.95)	< 0.001***
Association of educational level with VI				
- Secondary level	0.44 (0.22–0.88)	0.020**	0.19 (0.06–0.53)	0.002***
- Non-degree level	0.20 (0.07–0.56)	0.003***	0.22 (0.03–0.89)	0.058*
- Degree level	0.21 (0.06–0.61)	0.006***	0.27 (0.04–1.11)	0.106
Association of age and educational level with VI				
-Age	1.05 (1.01–1.09)	0.018**	1.07 (1.01–1.14)	0.030**
-Educational level				
- Secondary level	0.55 (0.27–1.13)	0.101	0.25 (0.08–0.74)	0.014**
- Non-degree level	0.26 (0.08–0.72)	0.013**	0.32 (0.05–1.40)	0.169
- Degree level	0.29 (0.08–0.86)	0.034**	0.44 (0.06–2.02)	0.333
Magnitude of confounding: relative change in OR of educational level on VI after adjusting the age				
- Secondary level	25%	(> 10%)	31.58%	(> 10%)
- Non-degree level	30%	(> 10%)	45.45%	(> 10%)
- Degree level	38%	(> 10%)	62.96%	(> 10%)

*CI* confidence interval, *VI* visual impairment

* *p*-value < 0.1; **p-value < 0.05; *** *p*-value < 0.01

In Table 3, we examined whether hyperlipidemia interacts with age, gender, obesity, hypertension or cataract on the risk for unilateral or bilateral VI. For unilateral VI, the interaction term between:

1. gender and a history of hyperlipidemia was significant (OR 0.14; 95% CI 0.02–0.71), implying that the risk effect of having a history of hyperlipidemia in females was significantly lower than in males.
2. obesity and a history of hyperlipidemia was significant (OR 17.64; 95% CI 2.82–164.9), implying that the risk effect of having a history of hyperlipidemia in obese subjects was significantly higher than in non-obese subjects.

**Table 3:** Multiplicative interaction model for observing whether hyperlipidemia interacts with age, gender, obesity, hypertension or cataract on the risk for unilateral and bilateral VI

	Unilateral VI		Bilateral VI	
	Est. (95% CI)	p-value	Est. (95% CI)	*p*-value
Model A				
History of hyperlipidemia	0.00 (0.00–18.45)	0.245	0.01 (0.00–304.4)	0.413
Age	1.05 (1.01–1.09)	0.012**	1.07 (1.00–1.14)	0.037**
History of hyperlipidemia: Age	1.09 (0.96–1.26)	0.217	1.08 (0.94–1.28)	0.308
Model B				
History of hyperlipidemia	3.55 (1.25–10.51)	0.019**	5.82 (1.54–23.11)	0.009***
Gender	1.29 (0.68–2.51)	0.444	1.02 (0.34–3.45)	0.971
History of hyperlipidemia: Gender	0.14 (0.02–0.71)	0.023**	0.15 (0.01–1.45)	0.142
Model C				
History of hyperlipidemia	0.52 (0.14–1.51)	0.266	1.81 (0.26–8.16)	0.477
Obesity	1.28 (0.65–2.48)	0.476	2.70 (0.88–8.30)	0.077*
History of hyperlipidemia: Obesity	17.64 (2.82–164.9)	0.005***	2.64 (0.33–27.57)	0.376
Model D				
History of hyperlipidemia	0.80 (0.17–2.81)	0.746	1.18 (0.06–7.26)	0.883
History of hypertension	1.69 (0.90–3.19)	0.104	1.39 (0.44–4.18)	0.559
History of hyperlipidemia: History of hypertension	2.17 (0.43–13.14)	0.365	3.45 (0.36–81.31)	0.332
Model E				
History of hyperlipidemia	1.02 (0.39–2.46)	0.965	2.75 (0.70–9.26)	0.116
History of cataract	1.23 (0.59–2.50)	0.574	1.99 (0.58–6.12)	0.242
History of hyperlipidemia: History of cataract	5.49 (0.87–48.40)	0.086*	1.66 (0.19–14.45)	0.640

*CI* confidence interval, *Est.* estimate, *VI* visual impairment

* *p*-value < 0.1; **p-value < 0.05; *** *p*-value < 0.01

Gender and obesity were likely to modify the association between hyperlipidemia and the risk for unilateral VI, so combining them would allow for a more accurate and greater effect than the sum of their individual effects. No significant interaction term was found for bilateral VI. We have summarized the non-significant results of examining the effect modification between variables on the risk for unilateral or bilateral VI. (Supplementary files 6–9. Tables 11, 12, 13, 14).

ORs, stratified by gender, obesity, hypertension, hyperlipidemia and cataract, were shown in Table 4. For unilateral VI, in stratified analysis, we found that:

1. stratified by gender, males with a history of hyperlipidemia (OR 3.55; 95% CI 1.25–10.51) and females with obesity (OR 2.44; 95% CI 1.09–5.48) suffered higher risk.
2. stratified by obesity, obese subjects having a history of hyperlipidemia (OR 9.21; 95% CI 2.15–64.1) and non-obese subjects having a history of hypertension (OR 2.18; 95% CI 1.04–4.53) suffered higher risk.
3. stratified by a history of hyperlipidemia, among those having a history, females (OR 0.18; 95% CI 0.03–0.79) suffered lower risk but subjects being obese (OR 22.5; 95% CI 4.11–192.5) and having a history of cataract (OR 6.75; 95% CI 1.25–53.54) suffered higher risk.
4. stratified by a history of cataract, subjects with a history of hyperlipidemia (OR 5.6; 95% CI 1.13–41.57) among those with a history and obese subjects (OR 2.08; 95% CI 1.05–4.14) among those without a history, suffered higher risk.

**Table 4:** Stratified analysis of odds ratios of gender, obesity, hypertension, hyperlipidemia and cataract on unilateral and bilateral visual impairment among 222 subjects

		Unilateral Visual Impairment		Bilateral Visual Impairment		Unilateral Visual Impairment		Bilateral Visual Impairment	
		Estimate (95% CI)	*p*-value	Estimate (95% CI)	*p*-value	Estimate (95% CI)	*p*-value	Estimate (95% CI)	*p*-value
A	Stratified by gender	Male				Female			
i	Obesity	1.53 (0.61–3.81)	0.359	5.78 (1.52–28.18)	0.015**	2.44 (1.09–5.48)	0.030**	2.11 (0.51–7.89)	0.271
ii	History of hypertension	2.21 (0.90–5.62)	0.087*	2.96 (0.79–14.32)	0.129	1.83 (0.85–3.90)	0.118	1.42 (0.35–5.24)	0.605
iii	History of hyperlipidemia	3.55 (1.25–10.51)	0.019**	5.82 (1.54–23.11)	0.009***	0.50 (0.11–1.68)	0.304	0.88 (0.05–5.23)	0.908
iv	History of cataract	2.29 (0.79–6.68)	0.125	4.04 (1.03–15.49)	0.040**	1.31 (0.56–2.96)	0.523	1.36 (0.28–5.22)	0.672
B	Stratified by obesity	Obesity				Non-obesity			
i	Gender	1.30 (0.49–3.48)	0.599	0.40 (0.10–1.43)	0.172	0.82 (0.40–1.70)	0.580	1.09 (0.28–5.35)	0.901
ii	History of hypertension	1.12 (0.41–3.06)	0.830	2.23 (0.59–10.87)	0.267	2.18 (1.04–4.53)	0.037**	1.13 (0.23–4.50)	0.864
iii	History of hyperlipidemia	9.21 (2.15–64.10)	0.007***	4.80 (1.16–19.81)	0.028**	0.52 (0.14–1.51)	0.266	1.81 (0.26–8.16)	0.477
iv	History of cataract	1.64 (0.48–5.76)	0.424	1.47 (0.29–6.02)	0.611	1.71 (0.77–3.71)	0.176	4.32 (1.08–18.33)	0.037**
C	Stratified by history of hypertension	Having a history				No history			
i	Gender	0.91 (0.39–2.13)	0.829	0.45 (0.11–1.56)	0.222	1.10 (0.49–2.59)	0.818	0.94 (0.24–4.64)	0.934
ii	Obesity	1.25 (0.53–2.94)	0.608	4.35 (1.19–20.82)	0.037**	2.43 (0.98–5.97)	0.052*	2.22 (0.44–9.08)	0.284
iii	History of hyperlipidemia	1.73 (0.64–4.73)	0.274	4.07 (1.13–14.82)	0.030**	0.80 (0.17–2.81)	0.746	1.18 (0.06–7.26)	0.883
iv	History of cataract	1.99 (0.81–5.02)	0.137	2.45 (0.70–8.67)	0.154	0.95 (0.32–2.54)	0.926	1.41 (0.20–6.36)	0.678
D	Stratified by history of hyperlipidemia	Having a history				No history			
i	Gender	0.18 (0.03–0.79)	0.032**	0.15 (0.01–1.08)	0.104	1.29 (0.68–2.51)	0.444	1.02 (0.34–3.45)	0.971
ii	Obesity	22.50 (4.11–192.5)	0.001***	7.14 (1.24–58.63)	0.038**	1.28 (0.65–2.48)	0.476	2.70 (0.88–8.30)	0.077*
iii	History of hypertension	3.67 (0.84–20.04)	0.100	4.80 (0.69–97.27)	0.172	1.69 (0.90–3.19)	0.104	1.39 (0.44–4.18)	0.559
iv	History of cataract	6.75 (1.25–53.54)	0.038**	3.30 (0.52–20.41)	0.190	1.23 (0.59–2.50)	0.574	1.99 (0.58–6.12)	0.242
E	Stratified by cataract	Having a history				No history			
i	Gender	0.57 (0.18–1.84)	0.346	0.26 (0.05–1.22)	0.093*	1.00 (0.52–1.95)	0.995	0.77 (0.25–2.50)	0.658
ii	Obesity	2.00 (0.56–7.40)	0.286	1.98 (0.36–9.62)	0.402	2.08 (1.05–4.14)	0.036**	5.83 (1.80–22.42)	0.005***
iii	History of hypertension	3.27 (1.03–11.40)	0.051*	2.86 (0.58–21.07)	0.227	1.56 (0.79–3.06)	0.192	1.65 (0.51–5.21)	0.388
iv	History of hyperlipidemia	5.60 (1.13–41.57)	0.049**	4.56 (0.76–25.60)	0.082*	1.02 (0.39–2.46)	0.965	2.75 (0.70–9.26)	0.116

*CI* confidence interval

* *p*-value < 0.1; **p-value < 0.05; *** *p*-value < 0.01

For bilateral VI, our results of stratified analysis showed that:

1. stratified by gender, males with obesity (OR 5.78; 95% CI 1.52–28.18), a history of hyperlipidemia (OR 5.82; 95% CI 1.54–23.11) and cataract (OR 4.04; 95% CI 1.03–15.49) suffered higher risk.
2. stratified by obesity, obese subjects having a history of hyperlipidemia (OR 4.8; 95% CI 1.16–19.81) and non-obese subjects having a history of cataract (OR 4.32; 95% CI 1.08–18.33) suffered higher risk.
3. stratified by a history of hypertension, among those having a history, subjects being obese (OR 4.35; 95% CI 1.19–20.82) and having a history of hyperlipidemia (OR 4.07; 95% CI 1.13–14.82), suffered higher risk.
4. stratified by a history of hyperlipidemia, obese subjects (OR 7.14; 95% CI 1.24–58.63), among those having a history, suffered higher risk.
5. stratified by a history of cataract, obese subjects (OR 5.83; 95% CI 1.8–22.42), among those not having a history, suffered higher risk.

### Eye-level analysis

On analysis of individual eyes, in Table 5, the proportions of myopia or hyperopia, abnormal or suspicious optic disc, and abnormal or undetermined macular appearance were 78.38, 13.51, 8.56 and 74.77%, 11.71, 9.01%, for the right and left eyes, respectively. VI was significantly associated with myopia or hyperopia (OR 6.69; 95% CI 2.07–21.60) and marginally associated with abnormal or undetermined macular appearance (OR 3.02; 95% CI 0.92–9.94) after adjusting the inter-eye correlation, but was not statistically associated with abnormal or suspicious optic disc. In the GLMM model for the association of VI with myopia or hyperopia, the variance estimates for random effects and intra-class correlation were 4.02 and 0.55 respectively to account for inter-eye correlation in outcome by adding a random effect.

**Table 5:** Associations between eye examination results and visual impairment at the eye-level analysis

	Descriptive statistics		Generalized linear mixed models	
	Eye examination results		Associations of VI with eye examination results	
	Right eye	Left eye	Estimate (95% CI)	*p*-value
	(*n* = 222)	(*n* = 222)		
Myopia or hyperopia				
No (Ref)	48 (21.62)	56 (25.23)	1.00	–
Yes	174 (78.38)	166 (74.77)	6.69 (2.07–21.60)	0.001***
Optic disc				
Normal cases (ref)	192 (86.49)	196 (88.29)	1.00	–
Abnormal or suspicious cases	30 (13.51)	26 (11.71)	0.81 (0.29–2.29)	0.697
Macula				
Normal cases (ref)	203 (91.44)	202 (81.98)	1.00	–
Abnormal or undetermined cases	19 (8.56)	20 (9.01)	3.02 (0.92–9.94)	0.069*

*CI* confidence interval, *VI* visual impairment

* *p*-value < 0.1; **p-value < 0.05; *** *p*-value < 0.01

## Discussion

The previous Hong Kong survey in the Shatin District by Lau et al. in 2002 found that 41.3% of the sampled population ≥ 60 had unilateral VI and 19.5% had bilateral VI. Our estimated age-and-sex-adjusted rates of subjects aged ≥60 in the Southern District in 2016 were 37.76% (95% CI, 28.76–48.69) for unilateral VI and 12.3% (95% CI, 7.39–19.24) for bilateral VI. For comparison, our results noted lower rate of bilateral VI, probably because the population composition of the two studies was different from the spatial (Shatin and Southern Districts) and temporal (2002 and 2016) perspectives of Hong Kong.

Our findings showed that sociodemographic disparities of visual health existed among our subjects. Chi Fu is generally considered a middle-class area in Hong Kong, but residents with older age, lower educational level, and living in temporary housing still had a higher risk for unilateral or bilateral VI. We did not find any significant association of VI with gender, marital status, employment status and household income. Previous studies in eastern China, Singapore and Korea found, in addition to older age and lower educational level, that being unemployed, separated, or females were independent risk factors for VI [34, 48, 49]. However, the associations of VI with gender, marital status and employment status have not been shown in other studies from southern India and Taiwan [50, 51].

In addition, we investigated the associations of VI with behavioral and medical risk factors. The results did not support any significant association of VI with smoking or alcohol-drinking histories but showed the significant correlation between bilateral VI and obesity or hyperlipidemia. Previous study showed that the association of VI with obesity may be bidirectional rather than unidirectional [52]. Obesity may have an indirect negative effect on VI due to the higher risk of vasculopathy which can affect blood vessels supplying the optic nerve and retina. Conversely, VI may be a causal factor for obesity as poor vision may hinder or limit outdoor activities and physical exercises.

ABI is a biomarker for peripheral artery disease (PAD), including the risks of heart attack, stroke, or poor peripheral circulation [42, 53]. To our knowledge, ABI has not been investigated for possible correlation with VI. We hypothesized that PAD is correlated with VI and investigated whether ABI would differ among older adults with VI compared to those with normal vision, assuming that poor blood circulation has potential negative effect on visual health. Our result did not support this hypothesis but this may be because our sample size was too small.

We did not find VI to be associated with the onsite blood pressure measurement, self-reported history of hypertension or DM. A possible explanation is that we only examined the presence or absence of DM and hypertension, but did not examine their duration or severity, and consider the threshold effect or dose-response relationship. One survey in eastern Taiwan showed that > 10 years disease duration of DM and hypertension were independently associated with VI, whereas 10 years or less of disease duration was not [54]. Another survey in the United States showed that higher level of LDL-cholesterol was correlated with a higher risk for blindness [31].

Although hyperlipidemia was not significantly associated with unilateral VI, interaction term between hyperlipidemia and gender or between hyperlipidemia and obesity was statistically significant. In stratified analysis, among those having a history of hyperlipidemia, female suffered lower risk for unilateral VI than male while obese subjects suffered higher risk than non-obese subjects. Gender and obesity were more likely to have multiplicative effect on unilateral VI when they are present with hyperlipidemia.

Gender, hypertension and cataract were not associated with unilateral or bilateral VI in general population of pooled analysis, but stratified analysis showed that females was associated with lower risk for unilateral VI in hyperlipidemia subgroup; having a history of hypertension was associated with higher risk for unilateral VI in non-obese subgroup; and having a history of cataract was associated with higher risk for unilateral VI in hyperlipidemia subgroup and bilateral VI in non-obese subgroup. Conversely, obesity and hyperlipidemia were associated with bilateral VI in general population of pooled analysis, but stratified analysis showed that subjects being obese and having a history of hyperlipidemia were not associated with higher risk for bilateral VI in female or cataract subgroup. The findings showed that ORs varied in specific subgroups of stratified analysis. Risk factors identified in the general population may not be applicable to specific subgroups. Inversely, non-significant risk factors in the general population may be applicable to specific subgroups.

From the eye-level analysis, the significant association of VI with refractive error (myopia or hyperopia) showed that uncorrected refractive error remains a significant cause of impaired vision in this population. The associations with abnormal, suspicious, or undetermined cases of macula and optic disc appearance were not significant, suggesting their unpredictable or inconsistent effect on VA. Similarly, subjects’ self-reported history of AMD, cataract or glaucoma was not significantly associated with VI. A possible explanation is that these were early cases or had received timely treatment for their condition which prevented significant visual loss. Another possible reason is that the sample size was small, resulting in wide confidence interval and biased results.

Our strength was that we investigated the visual health problems from a multi-perspective view. However, there were a few limitations. First, the sample size was relatively small, making subgroup analysis difficult. Second, since a free eye examination was provided, there may have been selection bias because people suffering from or interested in eye diseases were more likely to join our study. Third, the medical history was self-reported and may be affected by recall bias. Lastly, people with impaired ambulation from severe visual or physical disability may not have been assessed by our survey since we did not arrange household visits.

## Conclusions

Spatial and temporal differences on the prevalence of bilateral VI were noted between our results and those from the previous survey by Lau et al. in 2002. Inequity of visual health was present, although the sampled community is considered a predominantly middle-class suburb. Subjects with older age, lower educational level, and/or living in temporary housing were vulnerable groups having higher risk for unilateral or bilateral VI. Obesity and hyperlipidemia were medical risk factors contributing to bilateral VI. Age was the confounders of the unilateral VI-hypertension, unilateral VI-educational level and bilateral VI-educational level relationships. Gender and obesity were more likely to have multiplicative effect on unilateral VI when combined with hyperlipidemia. Stratified analysis should be conducted to provide further insight into the risk factors for VI in specific populations, in addition to general population. Refractive error (myopia or hyperopia) was associated with VI in the eye-level analysis. We recommend conducting a larger, territory-wide survey of the elderly population in Hong Kong, to better assess the overall prevalence of VI and the effects of varying geographical locations.

## Supplementary information

**Additional file 1: Table 6.** Sociodemographic Profiles of Population in Chi Fu DCCA, Southern District, Shatin District and Hong Kong.

**Additional file 2: Table 7.** Characteristics of the 222 respondents in the study.

**Additional file 3: Table 8.** Prevalence of low vision, blindness and visual impairment by the 222 respondents from a southern suburb of Hong Kong in January 2016.

**Additional file 4: Table 9.** Epidemiologic factors associated with visual impairment by the 222 respondents from a southern suburb of Hong Kong in January 2016: Univariate logistic regression.

**Additional file 5: Table 10.** Associations of behavioural factors with medical risk factors among the 222 respondents.

**Additional file 6: Table 11.** Multiplicative interaction model for observing whether gender interacts with age, obesity, hypertension, hyperlipidemia or cataract on the risk for unilateral and bilateral VI.

**Additional file 7: Table 12.** Multiplicative interaction model for observing whether obesity interacts with age, gender, hypertension, hyperlipidemia or cataract on the risk for unilateral and bilateral VI.

**Additional file 8: Table 13.** Multiplicative interaction model for observing whether hypertension interacts with age, gender, obesity, hyperlipidemia or cataract on the risk for unilateral and bilateral VI.

**Additional file 9: Table 14.** Multiplicative interaction model for observing whether cataract interacts with age, gender, obesity, hypertension or hyperlipidemia on the risk for unilateral and bilateral VI.

**Additional file 10:** Questionnaire (original). Original traditional Chinese version.

**Additional file 11:** Questionnaire (translated). Translated English version.

## Data Availability

The datasets and computing code used during the current study are available from the corresponding author on reasonable request.

## Abbreviations

ABI: Ankle-brachial index
AMD: Age-related macular degeneration
AORs: Adjusted odds ratios
BMI: Body Mass Index
CIs: Confidence intervals
CORs: Crude odds ratios
DCCAs: District Council Constituency Areas
DM: Diabetes mellitus
EC: Ethics Committees
GLMM: Generalized linear mixed effects models
IQR: Interquartile range
IRB: Institutional Review Board
LDL: Low-density lipoprotein
logMAR: Logarithm of the Minimum Angle of Resolution
OCT: Optical coherence tomography
ORs: Odds ratios
PAD: Peripheral artery disease
QoL: Quality of life
RNFL: Retinal nerve fiber layer
VA: Visual acuity
VI: Visual impairment
WHO: World Health Organization
