# Supplementary material for: Sociodemographic, behavioral, and medical risk factors associated with visual impairment among older adults: a community-based pilot survey in Southern District of Hong Kong
Source: BMC Ophthalmol. 2020 Sep 18;20:372. doi: 10.1186/s12886-020-01644-1 (PMC7501719; doi:10.1186/s12886-020-01644-1)
Supplement: Supplementary file 1 — Additional file 1: Table 6. Sociodemographic Profiles of Population in Chi Fu DCCA, Southern District, Shatin District and Hong Kong. [file 12886_2020_1644_MOESM1_ESM.docx]

| **Table 6. Sociodemographic Profiles of Population in Chi Fu DCCA, Southern District, Shatin District and Hong Kong** | | | | | | | | | | | | | | | | | |
| --- | --- | --- | --- | --- | --- | --- | --- | --- | --- | --- | --- | --- | --- | --- | --- | --- | --- |
|  |  |  |  |  |  |  |  |  |  |  |  |  |  |  |  |  |  |
|  |  |  | Chi Fu DCCA | | |  | Southern District | | |  | Shatin District | | |  | Hong Kong | | |
| Characteristics |  | Sub-groups | Median | Frequency (n) | Percentage (%) |  | Median | Frequency (n) | Percentage (%) |  | Median | Frequency (n) | Percentage (%) |  | Median | Frequency (n) | Percentage (%) |
|  |  |  |  |  |  |  |  |  |  |  |  |  |  |  |  |  |  |
| Number of overall population |  |  |  | 15,784 |  |  |  | 274,994 |  |  |  | 659,794 |  |  |  | 7,336,585 |  |
| Number of domestic households |  |  |  | 5,437 |  |  |  | 85,505 |  |  |  | 221,821 |  |  |  | 2,509,734 |  |
|  |  |  |  |  |  |  |  |  |  |  |  |  |  |  |  |  |  |
| Age (years) - Overall |  |  | 43.7 |  |  |  | 43.9 |  |  |  | 44.2 |  |  |  | 43.4 |  |  |
| Age (years) - Male |  |  | 45.4 |  |  |  | 44.3 |  |  |  | 44.6 |  |  |  | 43.7 |  |  |
| Age (years) - Female |  |  | 42.7 |  |  |  | 43.6 |  |  |  | 43.9 |  |  |  | 43.2 |  |  |
|  |  |  |  |  |  |  |  |  |  |  |  |  |  |  |  |  |  |
| Age group (years) |  | 50-59 |  | 2,826 | 43.97 |  |  | 47,354 | 42.44 |  |  | 114,379 | 43.46 |  |  | 1,266,114 | 43.29 |
| (extracting population aged ≥ 50) |  | 60-69 |  | 2,150 | 33.45 |  |  | 33,524 | 30.04 |  |  | 79,212 | 30.10 |  |  | 890,982 | 30.47 |
|  |  | 70-79 |  | 826 | 12.85 |  |  | 16,117 | 14.44 |  |  | 38,609 | 14.67 |  |  | 427,201 | 14.61 |
|  |  | ≥80 |  | 625 | 9.72 |  |  | 14,590 | 13.08 |  |  | 31,001 | 11.78 |  |  | 340,249 | 11.63 |
|  |  |  |  |  |  |  |  |  |  |  |  |  |  |  |  |  |  |
| Gender |  | Male |  | 2,990 | 46.52 |  |  | 51,944 | 46.55 |  |  | 124,193 | 47.19 |  |  | 1,386,704 | 47.42 |
| (extracting population aged ≥ 50) |  | Female |  | 3,437 | 53.48 |  |  | 59,641 | 53.45 |  |  | 139,008 | 52.81 |  |  | 1,537,842 | 52.58 |
|  |  |  |  |  |  |  |  |  |  |  |  |  |  |  |  |  |  |
| Educational level |  | Primary level or below |  | 1,402 | 10.02 |  |  | 51,756 | 21.23 |  |  | 110,437 | 18.88 |  |  | 1,299,920 | 19.98 |
| (extracting population aged ≥ 15) |  | Secondary level (incl. matriculation) |  | 5,547 | 39.64 |  |  | 105,284 | 43.19 |  |  | 269,719 | 46.11 |  |  | 3,076,103 | 47.28 |
|  |  | Non-degree level |  | 1,773 | 12.67 |  |  | 25,288 | 10.37 |  |  | 64,369 | 11.00 |  |  | 684,390 | 10.52 |
|  |  | Degree level |  | 5,273 | 37.68 |  |  | 61,428 | 25.20 |  |  | 140,471 | 24.01 |  |  | 1,445,717 | 22.22 |
|  |  |  |  |  |  |  |  |  |  |  |  |  |  |  |  |  |  |
| Housing type |  | Private permanent housing |  | 5,200 | 95.64 |  |  | 42,964 | 50.25 |  |  | 95,763 | 43.17 |  |  | 3,901,743 | 53.18 |
| (Number of domestic households) |  | Non-domestic, pubic or temporary housing |  | 237 | 4.36 |  |  | 42,541 | 49.75 |  |  | 126,058 | 56.83 |  |  | 3,434,842 | 46.82 |
|  |  |  |  |  |  |  |  |  |  |  |  |  |  |  |  |  |  |
| Marital status |  | Never married |  | 4,049 | 28.93 |  |  | 75,211 | 30.86 |  |  | 168,994 | 28.89 |  |  | 1,957,284 | 30.08 |
| (extracting population aged ≥ 15) |  | Married |  | 8,625 | 61.63 |  |  | 139,726 | 57.32 |  |  | 350,429 | 59.90 |  |  | 3,797,421 | 58.37 |
|  |  | Divorced/ Widowed/ Separated |  | 1,321 | 9.43 |  |  | 28,819 | 11.82 |  |  | 65,573 | 11.21 |  |  | 751,425 | 11.55 |
|  |  |  |  |  |  |  |  |  |  |  |  |  |  |  |  |  |  |
| Employment status |  | Employee/ employer/ self-employed/ unpaid family workers |  | 8,746 | 55.41 |  |  | 143,331 | 52.12 |  |  | 335,202 | 50.80 |  |  | 3,756,612 | 51.20 |
|  |  | Retired/ homemakers |  | 3,511 | 22.24 |  |  | 57,964 | 21.08 |  |  | 157,788 | 23.91 |  |  | 1,706,591 | 23.26 |
|  |  | Students |  | 2,211 | 14.01 |  |  | 40,014 | 14.55 |  |  | 103,750 | 15.72 |  |  | 1,098,542 | 14.97 |
|  |  | Others |  | 1,316 | 8.34 |  |  | 33,685 | 12.25 |  |  | 63,054 | 9.56 |  |  | 774,840 | 10.56 |
|  |  |  |  |  |  |  |  |  |  |  |  |  |  |  |  |  |  |
| Median monthly household income ($) |  |  | 39,160 |  |  |  | 30,000 |  |  |  | 27,180 |  |  |  | 25,000 |  |  |
|  |  |  |  |  |  |  |  |  |  |  |  |  |  |  |  |  |  |
| Monthly household income |  | Less than $10,000 |  | 795 | 14.62 |  |  | 12,955 | 15.15 |  |  | 40,581 | 18.29 |  |  | 480,117 | 19.13 |
| (Number of domestic households) |  | $10,000 - $24,999 |  | 972 | 17.88 |  |  | 22,995 | 26.89 |  |  | 62,153 | 28.02 |  |  | 768,719 | 30.63 |
|  |  | $25,000 or above |  | 3,670 | 67.50 |  |  | 49,555 | 57.96 |  |  | 119,087 | 53.69 |  |  | 1,260,898 | 50.24 |
|  |  |  |  |  |  |  |  |  |  |  |  |  |  |  |  |  |  |
| Source: 2016 Population By-census. Census and Statistics Department, Hong Kong Special Administrative Region Government | | | | | | | | | | | | | | | | | |
