# Supplementary material for: Sociodemographic, behavioral, and medical risk factors associated with visual impairment among older adults: a community-based pilot survey in Southern District of Hong Kong
Source: BMC Ophthalmol. 2020 Sep 18;20:372. doi: 10.1186/s12886-020-01644-1 (PMC7501719; doi:10.1186/s12886-020-01644-1)
Supplement: Supplementary file 2 — Additional file 2: Table 7. Characteristics of the 222 respondents in the study. [file 12886_2020_1644_MOESM2_ESM.docx]

| **Table 7. Characteristics of the 222 respondents in the study** | | | | | |
| --- | --- | --- | --- | --- | --- |
|  |  |  |  |  |  |
|  |  | Sub-groups | Median (IQR) | Frequency (n) | Percentage (%) |
|  |  |  |  |  |  |
| **Sociodemographic Characteristics** |  |  |  |  |  |
|  |  |  |  |  |  |
| Age (years) |  |  | 67 (61 - 72) |  |  |
|  |  |  |  |  |  |
| Age group (years) |  | 50-59 |  | 40 | 18.02 |
|  |  | 60-69 |  | 110 | 49.55 |
|  |  | 70-79 |  | 53 | 23.87 |
|  |  | ≥80 |  | 19 | 8.56 |
|  |  |  |  |  |  |
| Gender |  | Male |  | 87 | 39.19 |
|  |  | Female |  | 135 | 60.81 |
|  |  |  |  |  |  |
| Educational level |  | Primary level or below |  | 48 | 21.62 |
|  |  | Secondary level (incl. matriculation) |  | 114 | 51.35 |
|  |  | Non-degree level |  | 33 | 14.86 |
|  |  | Degree level |  | 27 | 12.16 |
|  |  |  |  |  |  |
| Housing type |  | Private permanent housing |  | 209 | 94.14 |
|  |  | Pubic or temporary housing |  | 13 | 5.86 |
|  |  |  |  |  |  |
| Marital status |  | Single |  | 10 | 4.50 |
|  |  | Married |  | 189 | 85.14 |
|  |  | Divorced/ Widowed |  | 23 | 10.36 |
|  |  |  |  |  |  |
| Employment status |  | Employed (full-time/ part-time) |  | 23 | 10.36 |
|  |  | Unemployed (retired/ job-seeker/ homemaker) |  | 199 | 89.64 |
|  |  |  |  |  |  |
| Monthly household income |  | $0 - $10,000 |  | 99 | 44.59 |
|  |  | $10,001 - $25,000 |  | 68 | 30.63 |
|  |  | $25,001 or above |  | 55 | 24.77 |
|  |  |  |  |  |  |
| **Behavioral factors** |  |  |  |  |  |
|  |  |  |  |  |  |
| Smoking |  | Non-smokers |  | 211 | 95.05 |
|  |  | Smokers |  | 3 | 1.35 |
|  |  | Ex-smokers |  | 8 | 3.60 |
|  |  |  |  |  |  |
| Drinking |  | Non-drinkers |  | 180 | 81.08 |
|  |  | Drinkers |  | 38 | 17.12 |
|  |  | Ex-drinkers |  | 4 | 1.80 |
|  |  |  |  |  |  |
| **Self-reported prevalence** |  |  |  |  |  |
|  |  |  |  |  |  |
| Diabetes mellitus (DM) |  |  |  | 28 | 12.61 |
| Hypertension |  |  |  | 88 | 39.64 |
| Hyperlipidemia |  |  |  | 34 | 15.32 |
| AMD |  |  |  | 9 | 4.05 |
| Cataract |  |  |  | 51 | 22.97 |
| Glaucoma |  |  |  | 6 | 2.70 |
|  |  |  |  |  |  |
| AMD, age-related macular degeneration; IQR, interquartile range | | | | | |
