# Supplementary material for: Sociodemographic, behavioral, and medical risk factors associated with visual impairment among older adults: a community-based pilot survey in Southern District of Hong Kong
Source: BMC Ophthalmol. 2020 Sep 18;20:372. doi: 10.1186/s12886-020-01644-1 (PMC7501719; doi:10.1186/s12886-020-01644-1)
Supplement: Supplementary file 3 — Additional file 3: Table 8. Prevalence of low vision, blindness and visual impairment by the 222 respondents from a southern suburb of Hong Kong in January 2016. [file 12886_2020_1644_MOESM3_ESM.docx]

| **Table 8. Prevalence of low vision, blindness and visual impairment by the 222 respondents from a southern suburb of Hong Kong in Jan 2016** | | | | | | | | | | | | | | |
| --- | --- | --- | --- | --- | --- | --- | --- | --- | --- | --- | --- | --- | --- | --- |
|  |  |  |  | Low vision | | |  | Blindness | | |  | Visual Impairment | | |
|  |  |  |  | n | % | (95% CI) |  | n | % | (95% CI) |  | n | % | (95% CI) |
| Right eye | |  |  |  |  |  |  |  |  |  |  |  |  |  |
|  | Crude |  |  | 41 | 18.47 | (13.25 - 25.05) |  | 5 | 2.25 | (0.73 - 5.26) |  | 46 | 20.72 | (15.17 - 27.64) |
|  | Age-and-sex adjusted | |  | 41 | 16.73 | (10.24 - 25.78) |  | 5 | 1.46 | (0.46 - 3.46) |  | 46 | 18.19 | (11.55 - 27.26) |
|  | Age-specific: | 50-59 |  | 4 | 10.0 | (3.25 - 24.6) |  | 0 | 0.00 | (0.00 - 10.91) |  | 4 | 10.0 | (3.25 - 24.6) |
|  |  | 60-69 |  | 18 | 16.36 | (10.24 - 24.9) |  | 3 | 2.73 | (0.71 - 8.35) |  | 21 | 19.09 | (12.46 - 27.93) |
|  |  | 70-79 |  | 12 | 22.64 | (12.73 - 36.55) |  | 2 | 3.77 | (0.66 - 14.08) |  | 14 | 26.42 | (15.68 - 40.58) |
|  |  | ≥80 |  | 7 | 36.84 | (17.23 - 61.37) |  | 0 | 0.00 | (0.00 - 20.92) |  | 7 | 36.84 | (17.23 - 61.37) |
|  | Gender-specific: | Male |  | 16 | 18.39 | (11.19 - 28.45) |  | 3 | 3.45 | (0.89 - 10.45) |  | 19 | 21.84 | (13.98 - 32.23) |
|  |  | Female |  | 25 | 18.52 | (12.56 - 26.32) |  | 2 | 1.48 | (0.26 - 5.79) |  | 27 | 20.0 | (13.81 - 27.94) |
| Left eye | |  |  |  |  |  |  |  |  |  |  |  |  |  |
|  | Crude |  |  | 45 | 20.27 | (14.79 - 27.12) |  | 3 | 1.35 | (0.28 - 3.95) |  | 48 | 21.62 | (15.94 - 28.67) |
|  | Age-and-sex adjusted | |  | 45 | 19.98 | (11.92 - 31.43) |  | 3 | 0.9 | (0.17 - 2.73) |  | 48 | 20.88 | (12.72 - 32.32) |
|  | Age-specific: | 50-59 |  | 3 | 7.5 | (1.96 - 21.48) |  | 0 | 0.00 | (0.00 - 10.91) |  | 3 | 7.5 | (1.96 - 21.48) |
|  |  | 60-69 |  | 24 | 21.82 | (14.74 - 30.91) |  | 0 | 0.00 | (0.00 - 4.21) |  | 24 | 21.82 | (14.74 - 30.91) |
|  |  | 70-79 |  | 11 | 20.75 | (11.29 - 34.5) |  | 2 | 3.77 | (0.66 - 14.08) |  | 13 | 24.53 | (14.19 - 38.58) |
|  |  | ≥80 |  | 7 | 36.84 | (17.23 - 61.37) |  | 1 | 5.26 | (0.28 - 28.11) |  | 8 | 42.11 | (21.12 - 66.03) |
|  | Gender-specific: | Male |  | 20 | 22.99 | (14.93 - 33.48) |  | 2 | 2.3 | (0.4 – 8.84) |  | 22 | 25.29 | (16.85 - 35.94) |
|  |  | Female |  | 25 | 18.52 | (12.56 - 26.32) |  | 1 | 0.74 | (0.04 – 4.67) |  | 26 | 19.26 | (13.18 - 27.13) |
| Bilateral | |  |  |  |  |  |  |  |  |  |  |  |  |  |
|  | Crude |  |  | 21 | 9.46 | (5.86 - 14.46) |  | 0 | 0.00 | (0.00 – 2.12) |  | 21 | 9.46 | (5.86 - 14.46) |
|  | Age-and-sex adjusted | |  | 21 | 6.89 | (4.14 - 10.78) |  | 0 | 0.00 | (0.00 – 2.12) |  | 21 | 6.89 | (4.14 - 10.78) |
|  | Age-specific: | 50-59 |  | 0 | 0.00 | (0.00 - 10.91) |  | 0 | 0.00 | (0.00 - 10.91) |  | 0 | 0.00 | (0.00 - 10.91) |
|  |  | 60-69 |  | 10 | 9.09 | (4.69 - 16.48) |  | 0 | 0.00 | (0.00 - 4.21) |  | 10 | 9.09 | (4.69 - 16.48) |
|  |  | 70-79 |  | 7 | 13.21 | (5.91 - 25.95) |  | 0 | 0.00 | (0.00 - 8.42) |  | 7 | 13.21 | (5.91 - 25.95) |
|  |  | ≥80 |  | 4 | 21.05 | (6.97 - 46.1) |  | 0 | 0.00 | (0.00 - 20.92) |  | 4 | 21.05 | (6.97 - 46.1) |
|  | Gender-specific: | Male |  | 11 | 12.64 | (6.78 - 21.91) |  | 0 | 0.00 | (0.00 - 5.27) |  | 11 | 12.64 | (6.78 - 21.91) |
|  |  | Female |  | 10 | 7.41 | (3.81 - 13.55) |  | 0 | 0.00 | (0.00 - 3.45) |  | 10 | 7.41 | (3.81 - 13.55) |
| Unilateral | |  |  |  |  |  |  |  |  |  |  |  |  |  |
|  | Crude |  |  | 65 | 29.28 | (22.6 - 37.32) |  | 8 | 3.6 | (1.56 - 7.1) |  | 73 | 32.88 | (25.77 - 41.35) |
|  | Age-and-sex adjusted | |  | 65 | 28.14 | (19.08 - 40) |  | 8 | 2.37 | (1 - 4.73) |  | 73 | 30.5 | (21.26 - 42.4) |
|  | Age-specific: | 50-59 |  | 7 | 17.5 | (7.89 - 33.36) |  | 0 | 0.00 | (0.00 - 10.91) |  | 7 | 17.5 | (7.89 - 33.36) |
|  |  | 60-69 |  | 32 | 29.09 | (21.02 - 38.65) |  | 3 | 2.73 | (0.71 - 8.35) |  | 35 | 31.82 | (23.45 - 41.48) |
|  |  | 70-79 |  | 16 | 30.19 | (18.74 - 44.51) |  | 4 | 7.55 | (2.45 - 19.07) |  | 20 | 37.74 | (25.12 - 52.13) |
|  |  | ≥80 |  | 10 | 52.63 | (29.5 - 74.79) |  | 1 | 5.26 | (0.28 - 28.11) |  | 11 | 57.89 | (33.97 - 78.88) |
|  | Gender-specific: | Male |  | 25 | 28.74 | (19.79 - 39.59) |  | 5 | 5.75 | (2.14 - 13.5) |  | 30 | 34.48 | (24.83 - 45.52) |
|  |  | Female |  | 40 | 29.63 | (22.24 - 38.2) |  | 3 | 2.22 | (0.58 - 6.86) |  | 43 | 31.85 | (24.25 - 40.5) |
| CI, confidence interval | | | | | | | | | | | | | | |
