# Supplementary material for: Sociodemographic, behavioral, and medical risk factors associated with visual impairment among older adults: a community-based pilot survey in Southern District of Hong Kong
Source: BMC Ophthalmol. 2020 Sep 18;20:372. doi: 10.1186/s12886-020-01644-1 (PMC7501719; doi:10.1186/s12886-020-01644-1)
Supplement: Supplementary file 4 — Additional file 4: Table 9. Epidemiologic factors associated with visual impairment by the 222 respondents from a southern suburb of Hong Kong in January 2016: Univariate logistic regression. [file 12886_2020_1644_MOESM4_ESM.docx]

| **Table 9. Epidemiologic factors associated with visual impairment by the 222 respondents from a southern suburb of Hong Kong in January 2016: Univariate logistic regression** | | | | | | | | | | | | | |
| --- | --- | --- | --- | --- | --- | --- | --- | --- | --- | --- | --- | --- | --- |
|  |  |  |  |  |  |  |  |  |  |  |  |  |  |
|  |  |  |  | Unilateral Visual Impairment | | | |  | Bilateral Visual Impairment | | | |  |
| **Variables** |  |  |  | Case (n=73) | Control (n=149) | COR (95% CI) | p-value | | Case (n=21) | Control (n=201) | COR (95% CI) | p-value | |
|  |  |  |  |  |  |  |  |  |  |  |  |  |  |
| Age (Years) |  | Median (IQR) |  | 68 (63 - 77) | 66 (60 - 70) | 1.06 (1.02 - 1.10) | 0.001 | *** | 72 (66 - 77) | 66 (61 - 71) | 1.09 (1.03 - 1.15) | 0.003 | *** |
|  |  |  |  |  |  |  |  |  |  |  |  |  |  |
| Gender: |  |  |  |  |  |  |  |  |  |  |  |  |  |
|  |  | Male (ref) |  | 30 (41.10) | 57 (38.26) | 1.00 | - |  | 11 (52.38) | 76 (37.81) | 1.00 | - |  |
|  |  | Female |  | 43 (58.90) | 92 (61.74) | 0.89 (0.50 - 1.58) | 0.684 |  | 10 (47.62) | 125 (62.19) | 0.55 (0.22 - 1.37) | 0.198 |  |
| Educational level: |  |  |  |  |  |  |  |  |  |  |  |  |  |
|  |  | Primary level or below (ref) |  | 25 (34.25) | 23 (15.44) | 1.00 | - |  | 11 (52.38) | 37 (18.41) | 1.00 | - |  |
|  |  | Secondary level |  | 37 (50.68) | 77 (51.68) | 0.44 (0.22 - 0.88) | 0.020 | ** | 6 (28.57) | 108 (53.73) | 0.19 (0.06 - 0.53) | 0.002 | *** |
|  |  | Non-degree level |  | 6 (8.22) | 27 (18.12) | 0.20 (0.07 - 0.56) | 0.003 | *** | 2 (9.52) | 31 (15.42) | 0.22 (0.03 - 0.89) | 0.058 | * |
|  |  | Degree level |  | 5 (6.85) | 22 (14.77) | 0.21 (0.06 - 0.61) | 0.006 | *** | 2 (9.52) | 25 (12.44) | 0.27 (0.04 - 1.11) | 0.106 |  |
| Housing type: |  |  |  |  |  |  |  |  |  |  |  |  |  |
|  |  | Private housing (ref) |  | 67 (91.78) | 142 (95.30) | 1.00 | - |  | 17 (80.95) | 192 (95.52) | 1.00 | - |  |
|  |  | Temporary housing |  | 6 (8.22) | 7 (4.70) | 1.82 (0.57 - 5.67) | 0.300 |  | 4 (19.05) | 9 (4.48) | 5.02 (1.26 - 17.26) | 0.013 | ** |
| Marital status: |  |  |  |  |  |  |  |  |  |  |  |  |  |
|  |  | Married (ref) |  | 62 (84.93) | 127 (85.23) | 1.00 | - |  | 21 (100.00) | 168 (83.58) | 1.00 | - |  |
|  |  | Single |  | 3 (4.11) | 7 (4.70) | 0.88 (0.18 - 3.28) | 0.854 |  | 0 (0.00) | 10 (4.98) | NA |  |  |
|  |  | Divorced/ Widowed |  | 8 (10.96) | 15 (10.07) | 1.09 (0.42 - 2.66) | 0.849 |  | 0 (0.00) | 23 (11.44) | NA |  |  |
| Employment status: |  |  |  |  |  |  |  |  |  |  |  |  |  |
|  |  | Employed (ref) |  | 6 (8.22) | 17 (11.41) | 1.00 | - |  | 2 (9.52) | 21 (10.45) | 1.00 | - |  |
|  |  | Unemployed |  | 67 (91.78) | 132 (88.59) | 1.44 (0.57 - 4.14) | 0.466 |  | 19 (90.48) | 180 (89.55) | 1.11 (0.29 - 7.26) | 0.895 |  |
| Monthly household income: |  |  |  |  |  |  |  |  |  |  |  |  |  |
|  |  | $0 - $10,000 (ref) |  | 38 (52.05) | 61 (40.94) | 1.00 | - |  | 12 (57.14) | 87 (43.28) | 1.00 | - |  |
|  |  | $10,001 - $25,000 |  | 24 (32.88) | 44 (29.53) | 0.88 (0.46 - 1.66) | 0.685 |  | 3 (14.29) | 65 (32.34) | 0.33 (0.07 - 1.10) | 0.100 |  |
|  |  | $25,001 or above |  | 11 (15.07) | 44 (29.53) | 0.40 (0.18 - 0.85) | 0.021 | ** | 6 (28.57) | 49 (24.38) | 0.89 (0.29 - 2.44) | 0.823 |  |
| Smoking: |  |  |  |  |  |  |  |  |  |  |  |  |  |
|  |  | Non-smokers (ref) |  | 69 (94.52) | 142 (95.30) | 1.00 | - |  | 19 (90.48) | 192 (95.52) | 1.00 | - |  |
|  |  | Smokers/ ex-smokers |  | 4 (5.48) | 7 (4.70) | 1.18 (0.30 - 4.03) | 0.801 |  | 2 (9.52) | 9 (4.48) | 2.25 (0.33 - 9.54) | 0.323 |  |
| Drinking: |  |  |  |  |  |  |  |  |  |  |  |  |  |
|  |  | Non-drinkers (ref) |  | 62 (84.93) | 118 (79.19) | 1.00 | - |  | 17 (80.95) | 163 (81.09) | 1.00 | - |  |
|  |  | Drinkers/ ex-drinkers |  | 11 (15.07) | 31 (20.81) | 0.68 (0.31 - 1.40) | 0.307 |  | 4 (19.05) | 38 (18.91) | 1.01 (0.28 - 2.91) | 0.987 |  |
| BMI |  |  |  |  |  |  |  |  |  |  |  |  |  |
|  |  | Normal (18.5 - 22.9) (ref) |  | 26 (35.62) | 66 (44.30) | 1.00 | - |  | 4 (19.05) | 88 (43.78) | 1.00 | - |  |
|  |  | Underweight (< 18.5) |  | 5 (6.85) | 7 (4.70) | 1.81 (0.50 - 6.20) | 0.345 |  | 2 (9.52) | 10 (4.98) | 4.40 (0.56 - 25.76) | 0.110 |  |
|  |  | Overweight (23 - 24.9) |  | 13 (17.81) | 39 (26.17) | 0.85 (0.38 - 1.81) | 0.673 |  | 3 (14.29) | 49 (24.38) | 1.35 (0.26 - 6.35) | 0.704 |  |
|  |  | Obese (≥ 25) |  | 29 (39.73) | 37 (24.83) | 1.99 (1.03 - 3.89) | 0.043 | ** | 12 (57.14) | 54 (26.87) | 4.89 (1.61 - 18.19) | 0.008 | *** |
| BMI ≥ 25 |  |  |  |  |  |  |  |  |  |  |  |  |  |
|  |  | No (Ref) |  | 44 (60.27) | 112 (75.17) | 1.00 | - |  | 9 (42.86) | 147 (73.13) | 1.00 | - |  |
|  |  | Yes |  | 29 (39.73) | 37 (24.83) | 2.00 (1.09 - 3.63) | 0.024 | ** | 12 (57.14) | 54 (26.87) | 3.63 (1.46 - 9.36) | 0.006 | *** |
| ABI |  |  |  |  |  |  |  |  |  |  |  |  |  |
|  |  | ≥ 1.0 and ≤ 1.4 (ref) |  | 58 (79.45) | 124 (83.22) | 1.00 | - |  | 16 (76.19) | 166 (82.59) | 1.00 | - |  |
|  |  | < 1.0 |  | 9 (12.33) | 18 (12.08) | 1.07 (0.43 - 2.47) | 0.879 |  | 4 (19.05) | 23 (11.44) | 1.80 (0.49 - 5.44) | 0.327 |  |
|  |  | > 1.4 |  | 6 (8.22) | 7 (4.70) | 1.83 (0.57 - 5.76) | 0.295 |  | 1 (4.76) | 12 (5.97) | 0.86 (0.05 - 4.84) | 0.892 |  |
|  |  |  |  |  |  |  |  |  |  |  |  |  |  |
| Systolic blood pressure |  | Median (IQR) |  | 133 (120 - 157) | 140 (125 - 153) | 1.00 (0.98 - 1.01) | 0.618 |  | 142 (125 - 155) | 138 (123 - 153) | 1.01 (0.99 - 1.03) | 0.446 |  |
|  |  |  |  |  |  |  |  |  |  |  |  |  |  |
| Diastolic blood pressure |  | Median (IQR) |  | 74 (65 - 81) | 75 (67 - 85) | 0.99 (0.97 - 1.01) | 0.241 |  | 76 (69 - 85) | 75 (66 - 84) | 1.01 (0.98 - 1.05) | 0.507 |  |
|  |  |  |  |  |  |  |  |  |  |  |  |  |  |
| Blood Pressure Category |  |  |  |  |  |  |  |  |  |  |  |  |  |
|  |  | Normal |  | 7 (9.59) | 16 (10.74) | 1.00 | - |  | 2 (9.52) | 21 (10.45) | 1.00 | - |  |
|  |  | Low Blood Pressure |  | 9 (12.33) | 10 (6.71) | 2.06 (0.59 - 7.54) | 0.264 |  | 0 (0.00) | 19 (9.45) | NA |  |  |
|  |  | Prehypertension |  | 29 (39.73) | 43 (28.86) | 1.54 (0.58 - 4.44) | 0.399 |  | 8 (38.10) | 64 (31.84) | 1.31 (0.30 - 9.15) | 0.743 |  |
|  |  | Hypertension |  | 28 (38.36) | 80 (53.69) | 0.80 (0.31 - 2.26) | 0.658 |  | 11 (52.38) | 97 (48.26) | 1.19 (0.29 - 8.06) | 0.828 |  |
| Diabetes mellitus |  |  |  |  |  |  |  |  |  |  |  |  |  |
|  |  | No (Ref) |  | 63 (86.30) | 131 (87.92) | 1.00 | - |  | 18 (85.71) | 176 (87.56) | 1.00 | - |  |
|  |  | Yes |  | 10 (13.70) | 18 (12.08) | 1.16 (0.49 - 2.61) | 0.733 |  | 3 (14.29) | 25 (12.44) | 1.17 (0.26 - 3.79) | 0.808 |  |
| Hypertension |  |  |  |  |  |  |  |  |  |  |  |  |  |
|  |  | No (Ref) |  | 36 (49.32) | 98 (65.77) | 1.00 | - |  | 9 (42.86) | 125 (62.19) | 1.00 | - |  |
|  |  | Yes |  | 37 (50.68) | 51 (34.23) | 1.97 (1.12 - 3.51) | 0.019 | ** | 12 (57.14) | 76 (37.81) | 2.19 (0.89 - 5.61) | 0.091 | * |
| Hyperlipidemia |  |  |  |  |  |  |  |  |  |  |  |  |  |
|  |  | No (Ref) |  | 59 (80.82) | 129 (86.58) | 1.00 | - |  | 14 (66.67) | 174 (86.57) | 1.00 | - |  |
|  |  | Yes |  | 14 (19.18) | 20 (13.42) | 1.53 (0.71 - 3.22) | 0.266 |  | 7 (33.33) | 27 (13.43) | 3.22 (1.13 - 8.51) | 0.021 | ** |
| AMD |  |  |  |  |  |  |  |  |  |  |  |  |  |
|  |  | No (Ref) |  | 68 (93.15) | 145 (97.32) | 1.00 | - |  | 20 (95.24) | 193 (96.02) | 1.00 | - |  |
|  |  | Yes |  | 5 (6.85) | 4 (2.68) | 2.67 (0.68 - 11.06) | 0.153 |  | 1 (4.76) | 8 (3.98) | 1.21 (0.06 - 7.08) | 0.863 |  |
| Cataract |  |  |  |  |  |  |  |  |  |  |  |  |  |
|  |  | No (Ref) |  | 52 (71.23) | 119 (79.87) | 1.00 | - |  | 13 (61.90) | 158 (78.61) | 1.00 | - |  |
|  |  | Yes |  | 21 (28.77) | 30 (20.13) | 1.60 (0.83 - 3.05) | 0.153 |  | 8 (38.10) | 43 (21.39) | 2.26 (0.85 - 5.73) | 0.090 | * |
| Glaucoma |  |  |  |  |  |  |  |  |  |  |  |  |  |
|  |  | No (Ref) |  | 69 (94.52) | 147 (98.66) | 1.00 | - |  | 20 (95.24) | 196 (97.51) | 1.00 | - |  |
|  |  | Yes |  | 4 (5.48) | 2 (1.34) | 4.26 (0.81 - 31.27) | 0.099 | * | 1 (4.76) | 5 (2.49) | 1.96 (0.10 - 12.97) | 0.548 |  |
| AMD/ Cataract/ Glaucoma |  |  |  |  |  |  |  |  |  |  |  |  |  |
|  |  | No (Ref) |  | 50 (68.49) | 116 (77.85) | 1.00 | - |  | 13 (61.90) | 153 (76.12) | 1.00 | - |  |
|  |  | Yes |  | 23 (31.51) | 33 (22.15) | 1.62 (0.86 - 3.02) | 0.133 |  | 8 (38.10) | 48 (23.88) | 1.96 (0.74 - 4.94) | 0.159 |  |
|  |  |  |  |  |  |  |  |  |  |  |  |  |  |
| AMD, age-related macular degeneration; COR, crude odds ratio; CI, confidence interval; NA, not applicable as odds ratio could not be calculated; IQR, interquartile range | | | | | | | | | | | | | |
| * p-value < 0.1; **p-value < 0.05; *** p-value < 0.01 | | | | | | | | | | | | | |
