# Supplementary material for: Sociodemographic, behavioral, and medical risk factors associated with visual impairment among older adults: a community-based pilot survey in Southern District of Hong Kong
Source: BMC Ophthalmol. 2020 Sep 18;20:372. doi: 10.1186/s12886-020-01644-1 (PMC7501719; doi:10.1186/s12886-020-01644-1)
Supplement: Supplementary file 5 — Additional file 5: Table 10. Associations of behavioural factors with medical risk factors among the 222 respondents. [file 12886_2020_1644_MOESM5_ESM.docx]

| Table 10. Associations of behavioural factors with medical risk factors among the 222 respondents | | | | | | | | |
| --- | --- | --- | --- | --- | --- | --- | --- | --- |
| Smoking | | | |  | Drinking | | | |
|  | COR (95% CI) | p-value | |  |  | COR (95% CI) | p-value | |
| Obesity (BMI ≥ 25) |  |  |  |  |  |  |  |  |
| Non-smokers (ref) | 1.00 | - |  |  | Non-drinkers (ref) | 1.00 | - |  |
| Smokers/ ex-smokers | 4.51 (1.31 - 17.74) | 0.020 | ** |  | Drinkers/ ex-drinkers | 1.23 (0.59 - 2.49) | 0.571 |  |
| History of diabetes mellitus | |  |  |  |  |  |  |  |
| Non-smokers (ref) | 1.00 | - |  |  | Non-drinkers (ref) | 1.00 | - |  |
| Smokers/ ex-smokers | 1.58 (0.23 - 6.57) | 0.571 |  |  | Drinkers/ ex-drinkers | 1.20 (0.42 - 3.01) | 0.717 |  |
| History of hypertension | |  |  |  |  |  |  |  |
| Non-smokers (ref) | 1.00 | - |  |  | Non-drinkers (ref) | 1.00 | - |  |
| Smokers/ ex-smokers | 17.05 (3.18 - 315.9) | 0.007 | *** |  | Drinkers/ ex-drinkers | 1.50 (0.76 - 2.95) | 0.242 |  |
| History of hyperlipidemia | |  |  |  |  |  |  |  |
| Non-smokers (ref) | 1.00 | - |  |  | Non-drinkers (ref) | 1.00 | - |  |
| Smokers/ ex-smokers | 1.24 (0.18 - 5.11) | 0.787 |  |  | Drinkers/ ex-drinkers | 1.13 (0.43 - 2.69) | 0.787 |  |
| History of AMD |  |  |  |  |  |  |  |  |
| Non-smokers (ref) | 1.00 | - |  |  | Non-drinkers (ref) | 1.00 | - |  |
| Smokers/ ex-smokers | NA |  |  |  | Drinkers/ ex-drinkers | 2.23 (0.46 - 8.85) | 0.271 |  |
| History of cataract |  |  |  |  |  |  |  |  |
| Non-smokers (ref) | 1.00 | - |  |  | Non-drinkers (ref) | 1.00 | - |  |
| Smokers/ ex-smokers | 1.99 (0.50 - 6.90) | 0.287 |  |  | Drinkers/ ex-drinkers | 0.62 (0.24 - 1.42) | 0.284 |  |
| History of glaucoma |  |  |  |  |  |  |  |  |
| Non-smokers (ref) | 1.00 | - |  |  | Non-drinkers (ref) | 1.00 | - |  |
| Smokers/ ex-smokers | 4.12 (0.20 - 28.98) | 0.215 |  |  | Drinkers/ ex-drinkers | 4.54 (0.81 - 25.33) | 0.070 | * |
| History of AMD, cataract or glaucoma | |  |  |  |  |  |  |  |
| Non-smokers (ref) | 1.00 | - |  |  | Non-drinkers (ref) | 1.00 | - |  |
| Smokers/ ex-smokers | 1.75 (0.44 - 6.03) | 0.388 |  |  | Drinkers/ ex-drinkers | 0.91 (0.40 - 1.94) | 0.815 |  |
| AMD, age-related macular degeneration; COR, crude odds ratio; CI, confidence interval | | | | | | | | |
| NA, not applicable as odds ratio could not be calculated; * p-value < 0.1; **p-value < 0.05; *** p-value < 0.01 | | | | | | | | |
