# Supplementary material for: Sociodemographic, behavioral, and medical risk factors associated with visual impairment among older adults: a community-based pilot survey in Southern District of Hong Kong
Source: BMC Ophthalmol. 2020 Sep 18;20:372. doi: 10.1186/s12886-020-01644-1 (PMC7501719; doi:10.1186/s12886-020-01644-1)
Supplement: Supplementary file 6 — Additional file 6: Table 11. Multiplicative interaction model for observing whether gender interacts with age, obesity, hypertension, hyperlipidemia or cataract on the risk for unilateral and bilateral VI. [file 12886_2020_1644_MOESM6_ESM.docx]

| Table 11. Multiplicative interaction model for observing whether gender interacts with age, obesity, hypertension, hyperlipidemia or cataract on the risk for unilateral and bilateral VI | | | | | | | | |
| --- | --- | --- | --- | --- | --- | --- | --- | --- |
|  |  | Unilateral VI | | |  | Bilateral VI | | |
|  |  | Est. (95% CI) | p-value |  |  | Est. (95% CI) | p-value |  |
| Model A |  |  |  |  |  |  |  |  |
| Gender |  | 0.60 (0.00 - 124.3) | 0.849 |  |  | 0.37 (0.00 - 1,515) | 0.811 |  |
| Age |  | 1.06 (0.99 - 1.12) | 0.069 | * |  | 1.08 (0.99 - 1.18) | 0.064 | * |
| Gender: Age |  | 1.01 (0.93 - 1.09) | 0.839 |  |  | 1.01 (0.90 - 1.13) | 0.889 |  |
| Model B |  |  |  |  |  |  |  |  |
| Gender |  | 0.82 (0.40 - 1.70) | 0.580 |  |  | 1.09 (0.28 - 5.35) | 0.901 |  |
| Obesity |  | 1.53 (0.61 - 3.81) | 0.359 |  |  | 5.78 (1.52 - 28.18) | 0.015 | ** |
| Gender: Obesity |  | 1.59 (0.47 - 5.39) | 0.452 |  |  | 0.37 (0.05 - 2.40) | 0.309 |  |
| Model C |  |  |  |  |  |  |  |  |
| Gender |  | 1.10 (0.49 - 2.59) | 0.818 |  |  | 0.94 (0.24 - 4.64) | 0.934 |  |
| History of hypertension |  | 2.21 (0.90 - 5.62) | 0.087 | * |  | 2.96 (0.79 - 14.32) | 0.129 |  |
| Gender: History of hypertension |  | 0.83 (0.25 - 2.68) | 0.752 |  |  | 0.48 (0.06 - 3.10) | 0.452 |  |
| Model D |  |  |  |  |  |  |  |  |
| Gender |  | 1.29 (0.68 - 2.51) | 0.444 |  |  | 1.02 (0.34 - 3.45) | 0.971 |  |
| History of hyperlipidemia |  | 3.55 (1.25 - 10.51) | 0.019 | ** |  | 5.82 (1.54 - 23.11) | 0.009 | *** |
| Gender: History of hyperlipidemia |  | 0.14 (0.02 - 0.71) | 0.023 | ** |  | 0.15 (0.01 - 1.45) | 0.142 |  |
| Model E |  |  |  |  |  |  |  |  |
| Gender |  | 1.00 (0.52 - 1.95) | 0.995 |  |  | 0.77 (0.25 - 2.50) | 0.658 |  |
| History of cataract |  | 2.29 (0.79 - 6.68) | 0.125 |  |  | 4.04 (1.03 - 15.49) | 0.040 | ** |
| Gender: History of cataract |  | 0.57 (0.15 - 2.19) | 0.415 |  |  | 0.34 (0.04 - 2.28) | 0.271 |  |
| CI, confidence interval; Est., estimate; VI, visual impairment | | | | | | | | |
| * p-value < 0.1; **p-value < 0.05; *** p-value < 0.01 | | | | | | | | |
