# Supplementary material for: Sociodemographic, behavioral, and medical risk factors associated with visual impairment among older adults: a community-based pilot survey in Southern District of Hong Kong
Source: BMC Ophthalmol. 2020 Sep 18;20:372. doi: 10.1186/s12886-020-01644-1 (PMC7501719; doi:10.1186/s12886-020-01644-1)
Supplement: Supplementary file 7 — Additional file 7: Table 12. Multiplicative interaction model for observing whether obesity interacts with age, gender, hypertension, hyperlipidemia or cataract on the risk for unilateral and bilateral VI. [file 12886_2020_1644_MOESM7_ESM.docx]

| Table 12. Multiplicative interaction model for observing whether obesity interacts with age, gender, hypertension, hyperlipidemia or cataract on the risk for unilateral and bilateral VI | | | | | | | | |
| --- | --- | --- | --- | --- | --- | --- | --- | --- |
|  |  | Unilateral VI | | |  | Bilateral VI | | |
|  |  | Est. (95% CI) | p-value |  |  | Est. (95% CI) | p-value |  |
| Model A |  |  |  |  |  |  |  |  |
| Obesity |  | 0.05 (0.00 - 19.63) | 0.339 |  |  | 10.94 (0.00 - 70,690) | 0.585 |  |
| Age |  | 1.05 (1.00 - 1.09) | 0.035 | ** |  | 1.10 (1.02 - 1.20) | 0.021 | ** |
| Obesity: Age |  | 1.06 (0.97 - 1.16) | 0.246 |  |  | 0.98 (0.87 - 1.11) | 0.797 |  |
| Model B |  |  |  |  |  |  |  |  |
| Obesity |  | 1.53 (0.61 - 3.81) | 0.359 |  |  | 5.78 (1.52 - 28.18) | 0.015 | ** |
| Gender |  | 0.82 (0.40 - 1.70) | 0.580 |  |  | 1.09 (0.28 - 5.35) | 0.901 |  |
| Obesity: Gender |  | 1.59 (0.47 - 5.39) | 0.452 |  |  | 0.37 (0.05 - 2.40) | 0.309 |  |
| Model C |  |  |  |  |  |  |  |  |
| Obesity |  | 2.43 (0.98 - 5.97) | 0.052 | * |  | 2.22 (0.44 - 9.08) | 0.284 |  |
| History of hypertension |  | 2.18 (1.04 - 4.53) | 0.037 | ** |  | 1.13 (0.23 - 4.50) | 0.864 |  |
| Obesity: History of hypertension |  | 0.51 (0.15 - 1.78) | 0.290 |  |  | 1.96 (0.28 - 17.01) | 0.511 |  |
| Model D |  |  |  |  |  |  |  |  |
| Obesity |  | 1.28 (0.65 - 2.48) | 0.476 |  |  | 2.70 (0.88 - 8.30) | 0.077 | * |
| History of hyperlipidemia |  | 0.52 (0.14 - 1.51) | 0.266 |  |  | 1.81 (0.26 - 8.16) | 0.477 |  |
| Obesity: History of hyperlipidemia |  | 17.64 (2.82 - 164.9) | 0.005 | *** |  | 2.64 (0.33 - 27.57) | 0.376 |  |
| Model E |  |  |  |  |  |  |  |  |
| Obesity |  | 2.08 (1.05 - 4.14) | 0.036 | ** |  | 5.83 (1.80 - 22.42) | 0.005 | *** |
| History of cataract |  | 1.71 (0.77 - 3.71) | 0.176 |  |  | 4.32 (1.08 - 18.33) | 0.037 | ** |
| Obesity: History of cataract |  | 0.96 (0.23 - 4.19) | 0.957 |  |  | 0.34 (0.04 - 2.45) | 0.293 |  |
| CI, confidence interval; Est., estimate; VI, visual impairment | | | | | | | | |
| * p-value < 0.1; **p-value < 0.05; *** p-value < 0.01 | | | | | | | | |
