# Supplementary material for: Sociodemographic, behavioral, and medical risk factors associated with visual impairment among older adults: a community-based pilot survey in Southern District of Hong Kong
Source: BMC Ophthalmol. 2020 Sep 18;20:372. doi: 10.1186/s12886-020-01644-1 (PMC7501719; doi:10.1186/s12886-020-01644-1)
Supplement: Supplementary file 8 — Additional file 8: Table 13. Multiplicative interaction model for observing whether hypertension interacts with age, gender, obesity, hyperlipidemia or cataract on the risk for unilateral and bilateral VI. [file 12886_2020_1644_MOESM8_ESM.docx]

| Table 13. Multiplicative interaction model for observing whether hypertension interacts with age, gender, obesity, hyperlipidemia or cataract on the risk for unilateral and bilateral VI | | | | | | | | |
| --- | --- | --- | --- | --- | --- | --- | --- | --- |
|  |  | Unilateral VI | | |  | Bilateral VI | | |
|  |  | Est. (95% CI) | p-value |  |  | Est. (95% CI) | p-value |  |
| Model A |  |  |  |  |  |  |  |  |
| History of hypertension |  | 0.02 (0.00 - 4.28) | 0.157 |  |  | 1.42 (0.00 - 6,298) | 0.935 |  |
| Age |  | 1.03 (0.98 - 1.08) | 0.311 |  |  | 1.08 (0.99 - 1.18) | 0.056 | * |
| History of hypertension: Age |  | 1.07 (0.99 - 1.16) | 0.116 |  |  | 1.00 (0.89 - 1.13) | 0.992 |  |
| Model B |  |  |  |  |  |  |  |  |
| History of hypertension |  | 2.21 (0.90 - 5.62) | 0.087 | * |  | 2.96 (0.79 - 14.32) | 0.129 |  |
| Gender |  | 1.10 (0.49 - 2.59) | 0.818 |  |  | 0.94 (0.24 - 4.64) | 0.934 |  |
| History of hypertension: Gender |  | 0.83 (0.25 - 2.68) | 0.752 |  |  | 0.48 (0.06 - 3.10) | 0.452 |  |
| Model C |  |  |  |  |  |  |  |  |
| History of hypertension |  | 2.18 (1.04 - 4.53) | 0.037 | ** |  | 1.13 (0.23 - 4.50) | 0.864 |  |
| Obesity |  | 2.43 (0.98 - 5.97) | 0.052 | * |  | 2.22 (0.44 - 9.08) | 0.284 |  |
| History of hypertension: Obesity |  | 0.51 (0.15 - 1.78) | 0.290 |  |  | 1.96 (0.28 - 17.01) | 0.511 |  |
| Model D |  |  |  |  |  |  |  |  |
| History of hypertension |  | 1.69 (0.90 - 3.19) | 0.104 |  |  | 1.39 (0.44 - 4.18) | 0.559 |  |
| History of hyperlipidemia |  | 0.80 (0.17 - 2.81) | 0.746 |  |  | 1.18 (0.06 - 7.26) | 0.883 |  |
| History of hypertension: History of hyperlipidemia |  | 2.17 (0.43 - 13.14) | 0.365 |  |  | 3.45 (0.36 - 81.31) | 0.332 |  |
| Model E |  |  |  |  |  |  |  |  |
| History of hypertension |  | 1.56 (0.79 - 3.06) | 0.192 |  |  | 1.65 (0.51 - 5.21) | 0.388 |  |
| History of cataract |  | 0.95 (0.32 - 2.54) | 0.926 |  |  | 1.41 (0.20 - 6.36) | 0.678 |  |
| History of hypertension: History of cataract |  | 2.09 (0.55 - 8.58) | 0.290 |  |  | 1.73 (0.24 - 16.66) | 0.599 |  |
| CI, confidence interval; Est., estimate; VI, visual impairment | | | | | | | | |
| * p-value < 0.1; **p-value < 0.05; *** p-value < 0.01 | | | | | | | | |
