# Supplementary material for: Sociodemographic, behavioral, and medical risk factors associated with visual impairment among older adults: a community-based pilot survey in Southern District of Hong Kong
Source: BMC Ophthalmol. 2020 Sep 18;20:372. doi: 10.1186/s12886-020-01644-1 (PMC7501719; doi:10.1186/s12886-020-01644-1)
Supplement: Supplementary file 9 — Additional file 9: Table 14. Multiplicative interaction model for observing whether cataract interacts with age, gender, obesity, hypertension or hyperlipidemia on the risk for unilateral and bilateral VI. [file 12886_2020_1644_MOESM9_ESM.docx]

| Table 14. Multiplicative interaction model for observing whether cataract interacts with age, gender, obesity, hypertension or hyperlipidemia on the risk for unilateral and bilateral VI | | | | | | | | |
| --- | --- | --- | --- | --- | --- | --- | --- | --- |
|  |  | Unilateral VI | | |  | Bilateral VI | | |
|  |  | Est. (95% CI) | p-value |  |  | Est. (95% CI) | p-value |  |
| Model A |  |  |  |  |  |  |  |  |
| History of cataract |  | 103.6 (0.11 - 94,661) | 0.181 |  |  | 119.7 (0.00 - 2,083,579) | 0.342 |  |
| Age |  | 1.08 (1.03 - 1.14) | 0.002 | *** |  | 1.11 (1.03 - 1.20) | 0.008 | *** |
| History of cataract: Age |  | 0.94 (0.85 - 1.03) | 0.172 |  |  | 0.94 (0.82 - 1.08) | 0.351 |  |
| Model B |  |  |  |  |  |  |  |  |
| History of cataract |  | 2.29 (0.79 - 6.68) | 0.125 |  |  | 4.04 (1.03 - 15.49) | 0.040 | ** |
| Gender |  | 1.00 (0.52 - 1.95) | 0.995 |  |  | 0.77 (0.25 - 2.50) | 0.658 |  |
| History of cataract: Gender |  | 0.57 (0.15 - 2.19) | 0.415 |  |  | 0.34 (0.04 - 2.28) | 0.271 |  |
| Model C |  |  |  |  |  |  |  |  |
| History of cataract |  | 1.71 (0.77 - 3.71) | 0.176 |  |  | 4.32 (1.08 - 18.33) | 0.037 | ** |
| Obesity |  | 2.08 (1.05 - 4.14) | 0.036 | ** |  | 5.83 (1.80 - 22.42) | 0.005 | *** |
| History of cataract: Obesity |  | 0.96 (0.23 - 4.19) | 0.957 |  |  | 0.34 (0.04 - 2.45) | 0.293 |  |
| Model D |  |  |  |  |  |  |  |  |
| History of cataract |  | 0.95 (0.32 - 2.54) | 0.926 |  |  | 1.41 (0.20 - 6.36) | 0.678 |  |
| History of hypertension |  | 1.56 (0.79 - 3.06) | 0.192 |  |  | 1.65 (0.51 - 5.21) | 0.388 |  |
| History of cataract: History of hypertension |  | 2.09 (0.55 - 8.58) | 0.290 |  |  | 1.73 (0.24 - 16.66) | 0.599 |  |
| Model E |  |  |  |  |  |  |  |  |
| History of cataract |  | 1.23 (0.59 - 2.50) | 0.574 |  |  | 1.99 (0.58 - 6.12) | 0.242 |  |
| History of hyperlipidemia |  | 1.02 (0.39 - 2.46) | 0.965 |  |  | 2.75 (0.70 - 9.26) | 0.116 |  |
| History of cataract: History of hyperlipidemia |  | 5.49 (0.87 - 48.40) | 0.086 | * |  | 1.66 (0.19 - 14.45) | 0.640 |  |
| CI, confidence interval; Est., estimate; VI, visual impairment | | | | | | | | |
| * p-value < 0.1; **p-value < 0.05; *** p-value < 0.01 | | | | | | | | |
