# Supplementary material for: Sociodemographic, behavioral, and medical risk factors associated with visual impairment among older adults: a community-based pilot survey in Southern District of Hong Kong
Source: BMC Ophthalmol. 2020 Sep 18;20:372. doi: 10.1186/s12886-020-01644-1 (PMC7501719; doi:10.1186/s12886-020-01644-1)
Supplement: Supplementary file 10 — Additional file 10: Questionnaire (original). Original traditional Chinese version. [file 12886_2020_1644_MOESM10_ESM.docx]

**香港大學李嘉誠醫學院眼科學系**

**南區公眾眼睛健康普查計劃**

Top of Form

個人資料

| 姓名： |  | | | | 性別： | 男 / 女 | | | 日期： |  |
| --- | --- | --- | --- | --- | --- | --- | --- | --- | --- | --- |
| 年齡： |  | 出生日期： | |  | | | | 身份證號碼： | |  |
| 聯絡電話(住宅) ： | | |  | | | | 其他聯絡電話： | | |  |
| 住址： | | | | | | | | | | |

背景資料

**請於答案的方格內加上🗹**

**慣用語言：** 🞏 廣東話 🞏 國語 🞏 英語 🞏 其他中國方言 🞏 其他: ___________

**教育程度：** 🞏 大學 (學位) 🞏 專上 (非學位) 🞏 中學 (包括預科程度) 🞏 小學

🞏 未受正規教育(識睇報紙及寫信) 🞏 未受教育/幼稚園

**居住房屋：** 🞏 公屋 🞏 居屋 🞏 私人房屋 🞏 臨時房屋 🞏 安老院舍 🞏 其他: ________

**婚姻狀況：** 🞏 未婚 🞏 已婚 🞏 同居 🞏 喪偶 🞏 已離婚

**現時職業：** 🞏 已退休 🞏 全職 🞏 兼職 🞏 待業 🞏 家庭主婦 🞏 其他: ________

**主要收入來源：**(可回答多過一項，最多三項，次序以最主要為先)

🞏 長俸/公積金 🞏 家人/親戚提供 🞏 薪金 🞏 綜合社會保障援助款項 (綜援)

🞏 傷殘津貼(DA) 🞏 高齡津貼(生果金) 🞏 積蓄 🞏 沒有收入 🞏 其他: ________

**家庭收入：** 🞏 $ 0 – $ 10,000 🞏 $ 10,001 – $ 25,000 🞏 $ 25,001或以上 🞏 不知道

生活習慣、個人病歷及家庭病歷

**吸煙狀況**

🞏 **不吸煙**

🞏 **吸煙** (請回答以下有關吸煙狀況之問題)

現時每日吸煙數量: 🞏 0-1包 🞏 1-2包 🞏 2-3包 🞏 3-4包 🞏 4包以上

吸煙年數: 🞏 0-5年 🞏 6-10年 🞏 10-15年 🞏 16-20年 🞏 20年以上

🞏 **已戒煙** (請回答以下有關戒煙狀況之問題)

戒煙前每日吸煙數量: 🞏 0-1包 🞏 1-2包 🞏 2-3包 🞏 3-4包 🞏 4包以上

吸煙年數: 🞏 0-5年 🞏 6-10年 🞏 10-15年 🞏 16-20年 🞏 20年以上

已戒煙年數: 🞏 0-5年 🞏 6-10年 🞏 10-15年 🞏 16-20年 🞏 20年以上

**飲酒狀況**

🞏 **不飲酒**

🞏 **飲酒** (請回答以下有關飲酒狀況之問題)

飲酒頻密程度： 🞏 甚少 🞏 間中 🞏 經常

現時每日飲酒數量: 🞏 0-2罐 🞏 3-4罐 🞏 5-6罐 🞏 7-8罐 🞏 8罐以上

飲酒年數: 🞏 0-5年 🞏 6-10年 🞏 10-15年 🞏 16-20年 🞏 20年以上

🞏 **已戒酒** (請回答以下有關戒酒狀況之問題)

戒酒前每日飲酒數量: 🞏 0-2罐 🞏 3-4罐 🞏 5-6罐 🞏 7-8罐 🞏 8罐以上

飲酒年數: 🞏 0-5年 🞏 6-10年 🞏 10-15年 🞏 16-20年 🞏 20年以上

已酒年數: 🞏 0-5年 🞏 6-10年 🞏 10-15年 🞏 16-20年 🞏 20年以上

**你曾否患有以下的慢性疾病呢？**

🞏 糖尿病 (一型／二型/ 不知道***** ) 🞏 高血壓 🞏 高血脂 🞏 以上皆沒有

**你曾否患有以下的眼部疾病呢？**

🞏 青光眼 🞏 白內障 🞏 老年黃斑病變 🞏 視網膜脫離 🞏 視網膜前膜

🞏 黃斑裂孔 🞏 糖尿視網膜病變 🞏 鼻淚管阻塞 🞏 葡萄膜炎 🞏 乾眼症

🞏 弱視 🞏 斜視 🞏 角膜炎 🞏 以上皆沒有

**你的*家人或朋友* 曾否患有以下的疾病呢？**

🞏 糖尿病 🞏 高血壓 🞏 高脂血 🞏 青光眼 🞏 白內障

🞏 老年黃斑病變 🞏 糖尿視網膜病變 🞏 以上皆沒有 🞏 其他：

對黃斑點疾病的認知

1. 你知道甚麼是黃斑點嗎？ 正常眼底視網膜組織 眼底視網膜致盲疾病 不知道
2. 你有聽過以下的黃斑點疾病嗎？
3. 老年黃斑病變 (AMD) 有 沒有
4. 糖尿病黃斑水腫 (DME) 有 沒有
5. 黃斑前膜 (ERM) 有 沒有
6. 黃斑全層裂孔 (FTMH) 有 沒有
7. 中漿病 (CSC) 有 沒有
8. 你知道知道老年黃斑病變會引致甚麼嗎？

中央視力模糊 周邊視力模糊 全盲 不知道

1. 你知道老年黃斑病變的自我檢測方法嗎？ 知道 不知道
2. 你知道老年黃斑病變的有效預防方法嗎？ 知道 不知道
3. 你知道老年黃斑病變有兩種類嗎？ 知道 不知道
4. 你知道以下哪一項是老年黃斑病變的先兆呢？ (可回答多過一項)

中央視力下降 周邊視力下降 影像變小及變形 影像變黃變暗

出現盲點 出現重影 出現光環 畏光眼痛

對顏色敏感度下降 眼痛 不知道 其他: ____________

1. 你知道老年黃斑病變會出現以下哪一項情況呢？(可回答多過一項)

眼底出血 晶體變白 眼底色素層萎縮 虹膜前移

眼底血管增生 視網膜脫落 出現玻膜疣 眼內液體未能吸收

眼底液體滲漏 眼壓過高 不知道 其他: ____________

1. 你知道以下哪一項是老年黃斑病變的治療方法呢？(可回答多過一項)

手術 口服藥物 激光治療 維他命補充劑

眼內注射 營養治療 光動力治療 中藥

佩戴矯視眼镜 不知道 其他: ___________________

對白內障的認知

1. 你有聽過白內障嗎？ 有 沒有
2. 你知道知道白內障會引致甚麼嗎？

中央視力模糊 周邊視力模糊 全盲 不知道

1. 你知道白內障的有效預防方法嗎？ 知道 不知道
2. 你知道以下哪一項是白內障的成因嗎？

藥物引致 由全身疾病引致 由其他眼睛疾病引致 年齡增長

外傷 眼睛感染發炎 先天 手術後引致

腦部受創後引致 不知道 其他，請說明: ___________________

1. 你知道以下哪一項是白內障的先兆呢？ (可回答多過一項)

中央視力下降 周邊視力下降 影像變小及變形 影像變黃變暗

出現盲點 出現重影 出現光環 畏光眼痛

對顏色敏感度下降 眼痛 不知道 其他: ____________

1. 你知道白內障會出現以下哪一項情況呢？(可回答多過一項)

眼底出血 晶體變白 眼底色素層萎縮 虹膜前移

眼底血管增生 視網膜脫落 出現玻膜疣 眼內導管閉塞

眼底液體滲漏 眼壓過高 不知道 其他: ____________

1. 你知道以下哪一項是白內障的治療方法呢？(可回答多過一項)

手術 口服藥物 激光治療 維他命補充劑

眼內注射 營養治療 光動力治療 中藥

佩戴矯視眼镜 不知道 其他，請說明: ___________________

對青光眼的認知

1. 你有聽過青光眼嗎？ 有 沒有
2. 你知道知道青光眼會引致甚麼嗎？

中央視力模糊 周邊視力模糊 全盲 不知道

1. 你知道青光眼的有效預防方法嗎？ 知道 不知道
2. 你知道青光眼分開角型和閉角型嗎？ 知道 不知道
3. 你知道以下哪一項是青光眼的成因嗎？

藥物引致 由全身疾病引致 由其他眼睛疾病引致 年齡增長

外傷 眼睛感染發炎 先天 手術後引致

腦部受創後引致 不知道 其他，請說明: ___________________

1. 你知道以下哪一項是青光眼的先兆呢？ (可回答多過一項)

中央視力下降 周邊視力下降 影像變小及變形 影像變黃變暗

出現盲點 出現重影 出現光環 畏光眼痛

對顏色敏感度下降 眼痛 不知道 其他: ____________

1. 你知道青光眼會出現以下哪一項情況呢？(可回答多過一項)

視野收窄 晶體變白 頭痛 虹膜前移

眼底血管增生 視網膜脫落 全盲 眼內導管閉塞

眼底液體滲漏 眼壓過高 不知道 其他: ____________

1. 你知道以下哪一項是青光眼的治療方法呢？(可回答多過一項)

手術 口服藥物 激光治療 維他命補充劑

眼內注射 營養治療 光動力治療 中藥

佩戴矯視眼镜 不知道 其他，請說明: ___________________
