# Supplementary material for: Sociodemographic, behavioral, and medical risk factors associated with visual impairment among older adults: a community-based pilot survey in Southern District of Hong Kong
Source: BMC Ophthalmol. 2020 Sep 18;20:372. doi: 10.1186/s12886-020-01644-1 (PMC7501719; doi:10.1186/s12886-020-01644-1)
Supplement: Supplementary file 11 — Additional file 11: Questionnaire (translated). Translated English version. [file 12886_2020_1644_MOESM11_ESM.docx]

A Hong Kong-wide epidemiological eye survey to study the prevalence, risk factors, and awareness of common eye diseases in Hong Kong

**Personal Information**

| Name: |  | | | | | Gender: | M / F | Date: | |  |
| --- | --- | --- | --- | --- | --- | --- | --- | --- | --- | --- |
| Age: |  | | Date of Birth: | |  | | | | HKID: |  |
| Telephone (Home): | | | |  | | | Telephone (Other): | | |  |
| Address: | |  | | | | | | | | |

**Please tick the appropriate box 🗹**

| \| 1. Usual language or dialects spoken \| \| --- \| \| 🞏 Cantonese \| \| 🞏 Putonghua \| \| 🞏 English \| \| 🞏 Other Chinese dialects \| \| 🞏 Others, please specify: __________________________________ \|  1. Please tell me your highest educational attainment |
| --- | --- | --- | --- | --- | --- | --- |
| 🞏 University level (Degree) |
| 🞏 Post-secondary level (Non-degree) |
| 🞏 Secondary level |
| 🞏 Primary level |
| 🞏 No formal education (Can read newspaper and write) |
| 🞏 No schooling/kindergarten |

| 1. What type of residence are you living in? |
| --- |
| 🞏 Public housing  🞏 Subsidized housing (Home Ownership Scheme) |
| 🞏 Private housing (self-purchased) |
| 🞏 Temporary housing |
| 🞏 Nursing home |
| 🞏 Others, please specify: __________________________________ |
| 1. Can you tell me your current marital status? |
| 🞏 Never married |
| 🞏 Married  🞏 Widowed |
| 🞏 Divorced |
|  |
| 1. Your current employment status is |
| 🞏 Retired |
| 🞏 Employed (Full-time) |
| 🞏 Employed (Part-time) |
| 🞏 Unemployed |
| 🞏 Homemaking |
| 🞏 Others, please specify: __________________________________ |

| 1. Your major source of income is (Up to three may be chosen in priority) |
| --- |
| 🞏 Pension / provident fund |
| 🞏 Family / relatives |
| 🞏 Salary |
| 🞏 Comprehensive Social Security Allowance (CSSA) |
| 🞏 Disability allowance (DA) |
| 🞏 Old age allowance (OA) |
| 🞏 Savings  🞏 No income |
| 🞏 Others, please specify: __________________________________ |

| 1. Your household income is |
| --- |
| 🞏 $ 0 – $ 10,000 |
| 🞏 $ 10,001 – $ 25,000 |
| 🞏 $ 25,001 or above |
| 🞏 Not known |

**Habits and Medical History**

Smoker: 🞏 Non-smoker

🞏 Current smoking 0-1 1-2 2-3 3-4 ≥4 packet/day

0-5 6-10 10-15 16-20  ≥20 years

🞏 Previous smoked 0-1 1-2 2-3 3-4 ≥4 packet/day

0-5 6-10 10-15 16-20 ≥20 years

- Stopped for 0-5 6-10 10-15 16-20 ≥20 years

Alcohol: 🞏 Non-drinker

🞏 Current drinker

- Frequency: 🞏 rarely 🞏 occasionally 🞏 regularly
- Daily intake: 🞏 0-2 🞏 3-4 🞏 5-6 🞏 7-8 🞏 ≥8 standard drinks
- Years of drinking: 0-5 6-10 10-15 16-20 ≥20

🞏 Previous Drinker

- Daily intake: 🞏 0-2 🞏 3-4 🞏 5-6 🞏 7-8 🞏 ≥8 standard drinks
- Years of drinking: 0-5 6-10 10-15 16-20 ≥20
- Stopped for (years): 0-5 6-10 10-15 16-20 ≥20

Have you ever been diagnosed with the following chronic diseases?

🞏 Diabetes Mellitus (Type I / Type II / Do not know)

🞏 Hypertension

🞏 Hyperlipidemia

Have you ever been diagnosed with the following eye diseases?

🞏 Glaucoma 🞏 Cataract 🞏 Age-related Macular Degeneration (AMD)

🞏 Retinal detachment 🞏 Epiretinal membrane 🞏 Macular holes 🞏 Diabetic retinopathy

🞏 Nasolacrimal duct obstruction 🞏 Uveitis 🞏 Dry eye disease 🞏 Amblyopia

🞏 Squint 🞏 Keratitis 🞏 None of above

**Family Medical History**

Have your family member ever suffered from the following eye disease?

🞏 Diabetes Mellitus 🞏 Hypertension 🞏 Hyperlipidemia

🞏 Glaucoma 🞏 Cataract 🞏 AMD 🞏 Diabetic retinopathy

🞏 Others: __________

**Knowledge related to Macular Diseases**

1. What is the “macula”? Part of the retina A retinal disease Do not know
2. Have you heard of the following macular diseases?
3. Age-related Macular Degeneration (AMD) Yes No
4. Diabetic Macular Edema (DME) Yes No
5. Epiretinal Membrane (ERM) Yes No
6. Full-Thickness Macular Hole (FTMH) Yes No
7. Central Serous Chorioretinopathy (CSC) Yes No
8. What is the consequence from AMD?

Central vision loss Peripheral vision loss Complete blindness Do not know

1. Do you know of any self-testing method for AMD? Yes No
2. Are you aware of any preventive measures for AMD? Yes No
3. Do you know the two types of AMD? Yes No
4. Which of the following(s) is/are the symptom(s) for AMD? (Can choose more than ONE answer)

Central vision loss Peripheral vision loss Deformed image Dim vision

New blind spot(s) Double vision Halo around lights Light sensitivity

Lower color sensitivity Ocular pain Do not know Others: ______

1. Which of the following(s) is/are the description(s) for AMD? (Can choose more than ONE answer)

Retinal hemorrhage Cloudy lens Degeneration of pigmented layer of retina

Forwardly placed iris Retinal vascular proliferation Retinal detachment

Drusen s Impaired aqueous humor outflow Retinal exudate

High intraocular pressure Do not know Others: ____________

1. Which of the following(s) is/are the treatment(s) for AMD? (Can choose more than ONE answer)

Surgery Oral medications Laser treatment

Vitamins supplement Intraocular injection Nutritional treatment

Photodynamic therapy Chinese (herbal) medicine Prescription glasses

Do not know Others: ____________

**Knowledge related to Cataract**

1. Have you heard of “cataract”? Yes No
2. What is the consequence from cataract?

Central vision loss Peripheral vision loss Complete blindness Do not know

1. Are you aware of any preventive measures for cataract? Yes No
2. Which of the following(s) is/are the cause(s) for cataract? (Can choose more than ONE answer)

Drug-induced Related to systemic diseases Related to other eye diseases

Aging Trauma Eye infections

Congenital Complication from surgery Brain injury

Do not know Others: ____________

1. Which of the following(s) is/are the symptom(s) for cataract? (Can choose more than ONE answer)

Central vision loss Peripheral vision loss Deformed image Dim vision

New blind spot(s) Double vision Halo around lights Light sensitivity

Lower color sensitivity Ocular pain Do not know Others: ______

1. Which of the following(s) is/are the description(s) for cataract? (Can choose more than ONE answer)

Retinal hemorrhage Cloudy lens Degeneration of pigmented layer of retina

Forwardly placed iris Retinal vascular proliferation Retinal detachment

Drusen s Impaired aqueous humor outflow Retinal exudate

High intraocular pressure Do not know Others: ____________

1. Which of the following(s) is/are the treatment(s) for cataract? (Can choose more than ONE answer)

Surgery Oral medications Laser treatment

Vitamins supplement Intraocular injection Nutritional treatment

Photodynamic therapy Chinese (herbal) medicine Prescription glasses

Do not know Others: ____________

**Knowledge related to Glaucoma**

1. Have you heard of “glaucoma”? Yes No
2. What is the consequence from glaucoma?

Central vision loss Peripheral vision loss Complete blindness Do not know

1. Are you aware of any effective method for preventing glaucoma? Yes No
2. Are you aware that glaucoma can be open-angle or closed-angle? Yes No
3. Which of the following(s) is/are the cause(s) for glaucoma? (Can choose more than ONE answer)

Drug-induced Related to systemic diseases Related to other eye diseases

Aging Trauma Eye infections

Congenital Complication from surgery Brain injury

Do not know Others: ____________

1. Which of the following(s) is/are the symptom(s) for glaucoma? (Can choose more than ONE answer)

Central vision loss Peripheral vision loss Deformed image Dim vision

New blind spot(s) Double vision Halo around lights Light sensitivity

Lower color sensitivity Ocular pain Do not know Others: ______

1. Which of the following(s) is/are the description(s) for glaucoma? (Can choose more than ONE answer)

Retinal hemorrhage Cloudy lens Degeneration of pigmented layer of retina

Forwardly placed iris Retinal vascular proliferation Retinal detachment

Drusen s Impaired aqueous humor outflow Retinal exudate

High intraocular pressure Do not know Others: ____________

1. Which of the following(s) is/are the treatment(s) for glaucoma? (Can choose more than ONE answer)

Surgery Oral medications Laser treatment

Vitamins supplement Intraocular injection Nutritional treatment

Photodynamic therapy Chinese (herbal) medicine Prescription glasses

Do not know Others: ____________

**~ END ~**
